# Novel Phenotypes of Acute Respiratory Failure and Differential Response to Awake Prone Positioning: A Multi‐Cohort Study

**DOI:** 10.1002/mco2.70818

**Published:** 2026-06-21

**Authors:** Nan Shi, Ruiqiang Zheng, Xufeng Chen, Huiying Zhao, Jun Jin, Changsong Wang, Shulin Xiang, Man Huang, Hongsheng Zhao, Yi Wang, Ruixuan Yu, Qin Sun, Hui Chen, Jianfeng Xie, Songqiao Liu, Yi Yang, Ling Liu, Haibo Qiu, the Chinese ARDS Research Network

**Affiliations:** ^1^ Department of Critical Care Medicine Jiangsu Provincial Key Laboratory of Critical Care Medicine Zhongda Hospital School of Medicine Southeast University Nanjing Jiangsu China; ^2^ Department of Critical Care Medicine Clinical Medical School Northern Jiangsu People's Hospital Yangzhou University Yangzhou Jiangsu China; ^3^ Department of Emergency Medicine Emergency Department Nanjing Medical University First Affiliated Hospital and Jiangsu Province Hospital Nanjing Jiangsu China; ^4^ Department of Critical Care Medicine Peking University People's Hospital Peking University Beijing China; ^5^ Department of Critical Care Medicine The First Affiliated Hospital of Soochow University Soochow University Suzhou Jiangsu China; ^6^ Department of Critical Care Medicine The First Affiliated Hospital of Harbin Medical University Harbin Heilongjiang China; ^7^ Department of Intensive Care Unit The Peoples Hospital of Guangxi Zhuang Autonomous Region Nanning Guangxi China; ^8^ General Intensive Care Unit Second Affiliated Hospital of Zhejiang University School of Medicine Hangzhou Zhejiang China; ^9^ Department of Critical Care Medicine Affiliated Hospital of Nantong University Nantong Jiangsu China; ^10^ Department of Critical Care Medicine The First Affiliated Hospital of Xinjiang Medical University Urumqi Xinjiang China; ^11^ The First People's Hospital of Lianyungang The Affiliated Lianyungang Hospital of Xuzhou Medical University The Lianyungang Clinical College of Nanjing Medical University Lianyungang Jiangsu China

**Keywords:** acute respiratory failure, awake prone positioning, phenotype

## Abstract

The heterogeneity of acute respiratory failure (ARF) in non‐intubated patients remains poorly defined. This study aimed to identify novel phenotypes in non‐intubated ARF patients and evaluate their prognostic and predictive ability. We analyzed data from three cohorts: a training cohort from the Chinese Database in Intensive Care, validation cohort A from a randomized controlled trial, and validation cohort B from a multicenter dataset. Latent class analysis and *k*‐means clustering were applied independently across cohorts to identify phenotypes. A two‐class model demonstrated the optimal fit in the training cohort (*n* = 706). Compared to Phenotype 1, Phenotype 2 exhibited worse oxygenation, higher lactate concentrations, and more severe coagulation dysfunction, along with significantly higher intubation (56.7 vs. 28.7%, *p *< 0.001) and mortality rates within 28 days (22.0 vs. 9.7%, *p *< 0.001). Similar phenotypes were verified in validation cohorts A (*n* = 409) and B (*n* = 609). Notably, Phenotype 1 patients benefited most from prolonged awake prone positioning (APP), with a significant interaction observed (*p* = 0.035; adjusted *p* = 0.048). Two parsimonious models were further developed for phenotype assignment prediction. The two novel phenotypes with both prognostic and predictive ability may help tailor APP to individual non‐intubated ARF patients.

## Graphical abstract

Two novel clinical phenotypes in non‐intubated patients with acute respiratory failure were identified and validated across three independent cohorts. Phenotype 2 was associated with worse oxygenation, coagulation dysfunction, higher lactate, and significantly higher intubation and mortality rates within 28 days compared to Phenotype 1. These phenotypes demonstrated prognostic and predictive ability, with Phenotype 1 patients benefiting most from prolonged awake prone positioning.

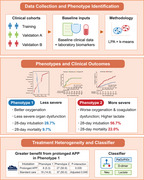

## 1 Introduction

Acute respiratory failure (ARF) is a common and heterogeneous clinical syndrome in intensive care unit with a high mortality [1, 2]. Patients with ARF exhibit variable manifestations and responses to treatments [3], suggesting the presence of distinct phenotypes that could contribute to individualized management approaches [4].

Although the phenotypes of intubated or mechanically ventilated ARF have been well established [5, 6, 7], few studies have explored phenotypes in non‐intubated patients. One notable study applied *k*‐means clustering among 41 patients receiving high‐flow nasal oxygen (HFNO) and identified two biological subphenotypes based on biomarkers associated with inflammation, lung epithelial damage, and endothelial injury [8]. Patients in the hyperinflammatory phenotype, similar to intubated cohorts, had worse outcomes. However, the study was limited by its small sample size, lack of validation, and unavailability of bedside testing. Another study focused on 85 COVID‐19 patients receiving oxygen therapy via face mask [9]. Three different clusters were identified by hierarchical clustering based mainly on inflammatory biomarkers. As disease severity and host response varied by baseline respiratory support level [10], whether patients receiving non‐intubated ventilatory support exhibit heterogeneity needed further investigation.

Awake prone positioning (APP), defined as an effective supportive intervention, has been reported to reduce the need for invasive mechanical ventilation in non‐intubated patients [11, 12]. A multicenter randomized controlled trial by Liu et al. demonstrated that prolonged APP significantly reduced intubation and mortality rate within 28 days in patients with COVID‐19‐related ARF [13]. However, whether specific phenotypes exist and respond more favorably to APP remain to clarify [14]. Recognizing the heterogeneity in response to APP is essential for optimizing its clinical use [15].

Given the limited understanding of phenotypes in non‐intubated ARF, this study aimed to identify novel phenotypes in non‐intubated ARF patients and evaluate whether these phenotypes could guide the application of APP therapy.

## 2 Results

### 2.1 Study Participants

A total of 706 patients were ultimately included in the training cohort of this study. The process of patient selection was shown in the Supporting Information. The median age was 68 years (interquartile range [IQR] 54–78), and 463 patients (65.6%) were male. The intubation rate was 33.7%, and the 28‐day mortality rate was 11.9%. The validation cohort A comprised 409 patients, with an intubation rate of 22.2% and a 28‐day mortality rate of 22.7%. In contrast, the validation cohort B consisted of 609 patients, with a 56.7% intubation and 37.8% mortality rate. Three cohorts differed significantly in their characteristics (Table S1). The schematic of study is outlined in Figure S1.

### 2.2 Identification of Phenotypes

**FIGURE 1:**
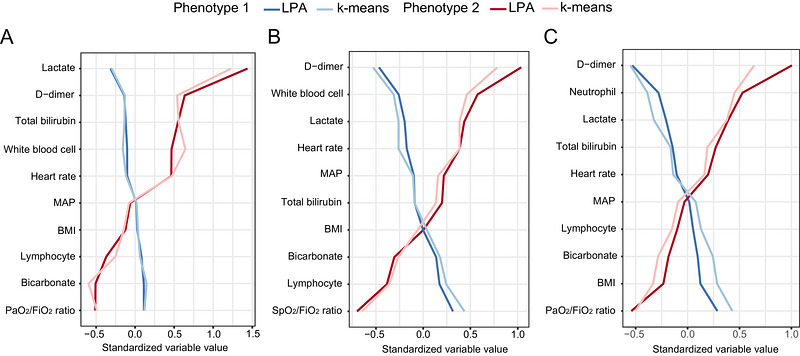
Comparison of variables that contributed to clinical phenotypes in three cohorts. (A) The training cohort. (B) The validation cohort A. (C) The validation cohort B. BMI, body‐mass index; LPA, latent profile analysis; MAP, mean arterial pressure.

Latent profile analysis (LPA) was applied to identify distinct clinical phenotypes among non‐intubated ARF patients, revealing that a two‐class model provided the best fit in the training cohort (Table S2). *K*‐means clustering produced similar results (Figure S2). Phenotype 1 (*n* = 579, 82.0%) and Phenotype 2 (*n* = 127, 18.0%) differed in clinical characteristics (Table 1 and Figure 1A). Compared to Phenotype 1, patients in Phenotype 2 were characterized with worse oxygenation, prolongation of prothrombin time, activated partial thromboplastin time, and higher D‐dimer and neutrophil counts but lower lymphocyte counts (*p* < 0.001 for all). Demographics, including age, sex distribution, and body‐mass index (BMI), were similar across phenotypes. The etiologies differed between the two phenotypes (*p* < 0.001). Phenotype 1 had a higher proportion of pneumonia and aspiration (58.0% vs. 34.7%), whereas Phenotype 2 was more frequently caused by sepsis (14.7% vs. 34.7%). In addition, Phenotype 2 presented more severe acidosis, renal, and hepatic dysfunction than Phenotype 1.

**TABLE 1:** Baseline characteristics, treatments, and outcomes of phenotypes using latent class analysis in the training cohort.

	All (n = 706)	Phenotype 1 (n = 579)	Phenotype 2 (n = 127)	*p* value
Age, median (IQR), years	68 (54, 78)	67 (53, 78)	70 (58, 79)	0.074
Sex (male), no. (%)	463 (65.6)	384 (66.3)	79 (62.2)	0.435
Body‐mass index, median (IQR), kg/m^2^	23.5 (20.8, 26.1)	23.7 (20.8, 26.2)	22.9 (20.8, 25.4)	0.111
Cause of ARF, no. (%)				<0.001
Pulmonary				
Pneumonia/Aspiration	380 (53.8)	336 (58.0)	44 (34.7)	
Contusion	19 (2.7)	17 (2.9)	2 (1.6)	
Extra‐pulmonary				
Sepsis	129 (18.3)	85 (14.7)	44 (34.7)	
Acute pancreatitis	78 (11.1)	62 (10.7)	16 (12.6)	
Others	100 (14.2)	79 (13.6)	21 (16.5)	
Coexisting illness, no. (%)				
Chronic heart disease	182 (25.8)	149 (25.7)	33 (26.0)	1.000
Hypertension	375 (53.1)	313 (54.1)	62 (48.8)	0.330
Diabetes	192 (27.2)	154 (26.6)	38 (29.9)	0.514
Chronic kidney disease	100 (14.2)	82 (14.2)	18 (14.2)	1.000
Chronic lung disease	93 (13.2)	82 (14.2)	11 (8.7)	0.130
Severe liver disease	15 (2.1)	9 (1.6)	6 (4.7)	0.037
Others	122 (17.3)	92 (15.9)	30 (23.6)	0.050
Vital signs and labs				
Heart rate, median (IQR), bpm	100 (97, 102)	100 (96, 101)	101 (100, 105)	<0.001
Mean arterial pressure, median (IQR), mmHg	75.0 (68.3, 84.7)	75.0 (68.7, 84.6)	75.0 (67.8, 86.2)	0.481
Platelet, median (IQR), ×10^9^/L	157.0 (103.0, 227.0)	164.5 (111.3, 239.8)	110.5 (69.8, 184.5)	<0.001
ALT, median (IQR), U/L	29.0 (16.0, 53.0)	27.5 (16.0, 48.3)	36.0 (19.0, 82.0)	0.001
AST, median (IQR), U/L	36.0 (22.0, 68.3)	32.0 (21.0, 58.0)	62.0 (28.5, 143.0)	<0.001
Total bilirubin, median (IQR), µmol/L	15.3 (9.4, 27.1)	13.5 (8.6, 24.1)	25.4 (13.8, 50.9)	<0.001
Creatinine, median (IQR), µmol/L	85.0 (61.0, 144.0)	79.0 (59.0, 133.5)	107.0 (72.0, 178.0)	<0.001
PaO_2_/FiO_2_ ratio, median (IQR)	183.1 (135.0, 222.2)	190.0 (143.9, 229.7)	150.5 (105.4, 191.6)	<0.001
FiO_2_, median (IQR), %	40 (37, 50)	40 (37, 49)	43 (39, 53)	0.001
White blood cell count, median (IQR), ×10^9^/L	10.9 (7.9, 14.5)	10.3 (7.7, 13.4)	13.5 (9.6, 19.3)	<0.001
Neutrophil, median (IQR), %	9.3 (6.4, 12.7)	8.9 (6.2, 11.8)	12.2 (8.0, 17.0)	<0.001
Lymphocyte count, median (IQR), ×10^9^/L	0.70 (0.44, 1.04)	0.74 (0.47, 1.07)	0.52 (0.32, 0.81)	<0.001
D‐dimer, median (IQR), µg/mL	1.9 (0.8, 3.1)	1.6 (0.7, 2.7)	3.0 (2.0, 5.3)	<0.001
PT, median (IQR), s	13.2 (12.0, 14.7)	13.0 (11.8, 14.3)	14.6 (12.9, 17.2)	<0.001
APTT, median (IQR), s	32.0 (28.7, 37.1)	31.4 (28.5, 35.9)	36.0 (30.6, 42.8)	<0.001
pH, median (IQR)	7.38 (7.34, 7.41)	7.38 (7.34, 7.41)	7.34 (7.3, 7.38)	<0.001
PaCO_2_, median (IQR), mmHg	41.5 (35.7, 47.7)	41.2 (35.7, 47.4)	43.4 (36.5, 49.7)	0.145
Bicarbonate, median (IQR), mmol/L	21.2 (18.4, 24.3)	21.7 (18.9, 24.5)	18.6 (16.1, 21.4)	<0.001
Lactate, median (IQR), mmol/L	2.0 (1.5, 2.7)	1.8 (1.4, 2.3)	4.2 (3.3, 5.1)	<0.001

Abbreviations: ALT, alanine aminotransferase; APTT, activated partial thromboplastin time; ARF, acute respiratory failure; AST, aspartate aminotransferase; IQR, interquartile range; LOS, length of stay; PT, prothrombin time.

Pairwise comparisons of variables in two validation cohorts revealed similarity between LPA and *k*‐means clustering, indicating good reproducibility of the model (Figure 1B,C, Tables S3 and S4, Figures S3 and S4). Overall, two clinically distinct phenotypes were consistently identified across all three cohorts, demonstrating robust reproducibility of the clustering approach.

### 2.3 Relationships Between Phenotypes and Clinical Outcomes

**FIGURE 2:**
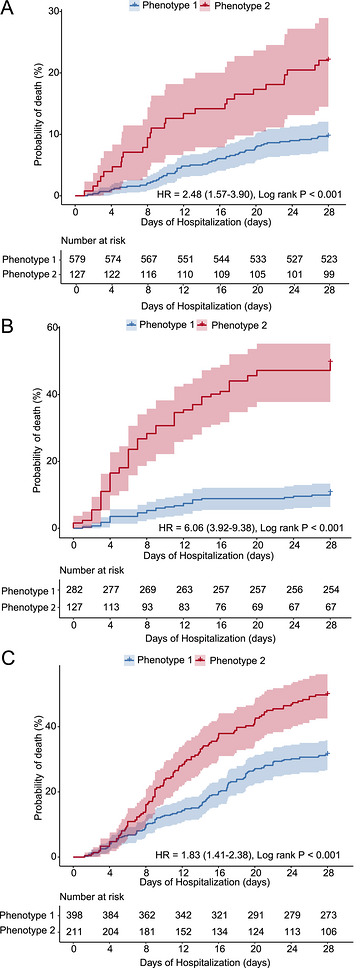
Probability of death by phenotypes. (A) The training cohort. (B) The validation cohort A. (C) The validation cohort B. Hazard ratio was calculated by Cox proportional‐hazards model. The log‐rank test demonstrated a significant between‐group difference. APP, awake prone positioning.

In the training cohort, patients in Phenotype 2 had significantly higher intubation rates (56.7% vs. 28.7%, *p* < 0.001) and 28‐day mortality rates (22.0% vs. 9.7%, *p* < 0.001) than those in Phenotype 1 (Table 2). Kaplan–Meier survival analysis and cumulative hazard calculations indicated worse 28‐day survival for Phenotype 2 patients (hazard ratio [HR] 2.48, 95% confidence interval [95% CI] 1.57–3.90, *p* < 0.001; Figure 2A). Similar trends were observed in the validation cohorts (Figure 2B,C and Table S5). These results highlighted the strong prognostic discriminative ability of the two phenotypes across independent cohorts.

**TABLE 2:** Treatment and outcomes of phenotypes in the training cohort.

	All (n = 706)	Phenotype 1 (n = 579)	Phenotype 2 (n = 127)	p value
Oxygenation mode on Day 1, no. (%)				
HFNO	653 (92.5)	533 (92.1)	120 (94.5)	0.449
NIV	129 (18.3)	110 (19)	19 (15)	0.347
Pharmacological intervention, no. (%)				
Glucocorticoids	335 (47.5)	248 (42.8)	87 (68.5)	<0.001
Thymosin	264 (37.4)	210 (36.3)	54 (42.5)	0.224
Anticoagulants	671 (95.0)	549 (94.8)	122 (96.1)	0.719
Vasopressor	402 (56.9)	294 (50.8)	108 (85.0)	<0.001
Intubation within 28 days, no. (%)	238 (33.7)	166 (28.7)	72 (56.7)	<0.001
Mortality at 28 days, no. (%)	84 (11.9)	56 (9.7)	28 (22.0)	<0.001
Intubation or death at 28 days, no. (%)	266 (37.7)	188 (32.5)	78 (61.4)	<0.001
Hospital LOS, day	18 (10, 29)	18 (10, 29)	18 (8, 28)	0.409

Abbreviations: HFNO, high‐flow nasal oxygen; LOS, length of stay; NIV, noninvasive ventilation.

### 2.4 Heterogeneity of the Treatment Effect

**FIGURE 3:**
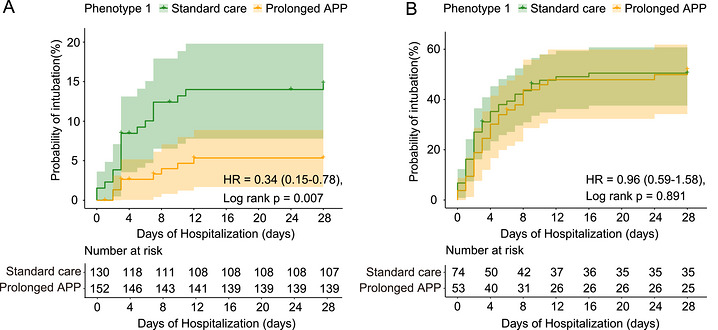
Probability of endotracheal intubation by awake prone positioning in the validation cohort A. (A) Phenotype 1. (B) Phenotype 2. Hazard ratio was calculated by Cox proportional‐hazards model. The log‐rank test demonstrated a significant between‐group difference. Prolonged APP: prolonged awake prone positioning, a minimum of 12 h daily with several breaks if needed for up to 7 days. Standard care: whether to be prone or not according to patients' wishes, however, they were not encouraged to remain in the prone position for a prolonged period of time (> 12 h/day). APP, awake prone positioning.

In the validation cohort A, the predictive ability of the phenotypes was examined. A significant interaction was observed between phenotype and the effect of prolonged APP strategy on 28‐day intubation rate (*p* = 0.035 for interaction; Table 3). After adjusted age, sex, and baseline SpO_2_/FiO_2_, the interaction *p* value was 0.048 (Table S6). Specifically, patients in Phenotype 1 who received prolonged APP had a significantly lower 28‐day intubation rate compared with control group (8/152 [5.3%] vs. 19/130 [14.6%], *p* = 0.014; Table S7). Cumulative incidence of 28‐day intubation within two phenotypes is shown in Figure 3.

**TABLE 3:** HTE of prolonged APP ventilation within phenotypes in the validation cohort A.

Outcomes	Phenotype 1 (n = 282)		Phenotype 2 (n = 127)		p value for interaction Adjusted p interaction	
	Prolonged APP (n = 152)	Standard care (n = 130)	Prolonged APP (n = 53)	Standard care (n = 74)		
Intubation within 28 days of randomization, no. (%)	8 (5.3)	19 (14.6)	27 (50.9)	37 (50.0)	0.035	0.048
Mortality at 28 days, no. (%)	11 (7.2)	19 (14.6)	27 (50.9)	36 (48.6)	0.114	0.160
Intubation or death at 28 days, no. (%)	13 (8.6)	24 (18.5)	29 (54.7)	39 (52.7)	0.061	0.090

*Note*: Adjusted for age, sex, and baseline SpO_2_/FiO_2_.

Abbreviations: APP, awake prone positioning; HTE, heterogeneity of the treatment effect.

### 2.5 Construction of a Phenotype Classifier

**FIGURE 4:**
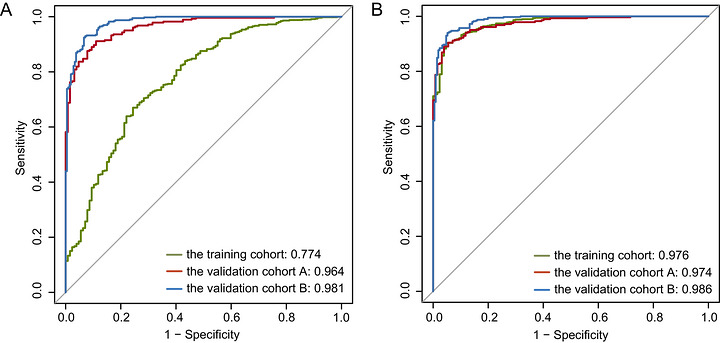
Predictability of different variables on the accuracy of classification of phenotypes on Day 1. (A) Three variables—PaO_2_/FiO_2_ ratio or SpO_2_/FiO_2_ ratio, neutrophil count or white blood cell count, D‐dimer. (B) Four variables—PaO_2_/FiO_2_ ratio or SpO_2_/FiO_2_ ratio, neutrophil count or white blood cell count, D‐dimer, lactate. Green curve: the training cohort, red curve: the validation cohort A, blue curve: the validation cohort B.

Two parsimonious models were developed for predicting the assignment of phenotypes. The first model incorporated three variables: The PaO_2_/FiO_2_ ratio, neutrophil count, and D‐dimer level, and the second model included lactate level as an additional variable. The receiver operating characteristic (ROC) curves showed superior predictive power for Phenotype 1 in both models, with area under the curves (AUCs) of 0.774 (95% CI: 0.927–0.821) and 0.976 (95% CI: 0.965–0.986) (Figure 4A, Tables S8 and S9). Likewise, these results were consistent in the validation cohorts (Figure 4B, Tables S10 and S11). Notably, the two parsimonious classifiers provided a practical tool for bedside phenotype identification.

## 3 Discussion

The main findings of this study can be summarized as follows. First, we identified and validated novel clinical phenotypes among non‐intubated patients with ARF. Second, the two phenotypes had different responses to prolonged APP strategy in the validation cohort A, with lower 28‐day intubation rate for prolonged APP used in Phenotype 1. Third, simplistic models were developed to predict non‐intubated phenotypes, enhancing the applicability at bedside.

Our phenotypes were partially consistent with those described in earlier work. Ranjeva et al. identified a unique phenotype characterized with hypercoagulability, end‐organ dysfunction, and severe lactic acidosis in intubated patients with acute respiratory distress syndrome (ARDS) [16]. Recognizing this parallel supported the idea that similar phenotypes existed even before intubation, at the stage of non‐intubated ARF. Identifying such phenotypes at this earlier point provided a valuable window for timely, individualized intervention and may ultimately help refine treatment thresholds.

These phenotypes differed from prior phenotypes in several critical aspects. First, different variables were applied for classification. Although previous studies have relied primarily on detailed, molecular inflammatory markers [5, 6, 7], we prioritized oxygenation and coagulation dysfunction. The priority can be interpreted in the context of the pathogenesis of acute lung injury (ALI) and ARDS, including alveolar epithelial injury, pulmonary vascular permeability, and intra‐alveolar fibrin deposition [1, 17]. D‐dimer, a recognized predictor of thrombosis and adverse outcomes, particularly in severe COVID‐19 [18, 19], has been shown to interact closely with inflammatory biomarkers in promoting vascular endothelial injury [20, 21]. Thus, the prognostic model identified PaO_2_/FiO_2_ ratio and D‐dimer as key variables in defining phenotypes apart from inflammatory factors. These results suggested D‐dimer may serve as a practical biomarker for future phenotypic differentiation and risk stratification [22]. Second, our analysis encompassed a broader population. We identified novel phenotypes in non‐intubated ARF patients irrespective of underlying causes, whereas some studies were limited to single disease cohort and lack of external validation [23, 24]. Patients in two COVID‐19 cohorts were from different regions and periods. Therefore, both the non‐COVID‐19 and COVID‐19 cohorts confirmed the existence and consistency of phenotypes in heterogeneous patient groups.

Several potential pathophysiologic mechanisms may account for the worse outcomes observed in Phenotype 2. In addition to oxygenation and coagulation dysfunction, and inflammatory responses, microcirculatory disturbances represented a critical contributing factor. High lactate, low pH, and low bicarbonate levels were notably pronounced in patients from the training cohort. Previous studies have demonstrated that lactate levels were independent predictors of mortality in ARF and ARDS [25, 26]. The underlying mechanism was that lactate accumulation impaired cardiac contractility, catecholamine responsiveness, and exacerbated tissue hypoperfusion [27]. Therefore, these findings also confirmed that no single clinical variable was sufficient to distinguish phenotype [28]. The four‐variable simplified model emphasized the feasibility of early bedside phenotyping to guide prolonged APP use and to inform the design of future therapeutic trials in non‐intubated ARF populations.

We also observed that patients with less severe disease derived greater benefit from prolonged APP. This finding was in line with subgroup analysis from the original trial. However, it contrasted with meta‐analyses suggesting greater benefit in patients with severe hypoxemia (SpO_2_/FiO_2_ < 150), or those receiving advanced noninvasive support [29, 30]. There are several possible explanations. First, disease severity is multidimensional and cannot be defined by respiratory support level alone; hypoxemia itself must be considered. Phenotype 1 marked by isolated respiratory failure may respond to a single supportive intervention such as APP, whereas severe or critical patients in Phenotype 2 probably required comprehensive management, in addition to respiratory support. Second, from a pathophysiological perspective, Yang et al. found that ARDS patients with focal lesions tended to have better oxygenation and a more rapid response to prone positioning [31]. This was also supported by the LIVE study indicating lung morphology‐guided treatments can reduce mortality [32]. The benefits were probably due to improved ventilation‐perfusion matching and compliance, reduced shunt, and lower PaCO_2_, all of which contributed to better oxygenation [31]. Thus, we hypothesized that less severe ARF patients with focal damages were more likely to benefit from prone positioning, although this remained unproven in COVID‐19‐related ARF [33]. Third, compared with those in Phenotype 2, a larger proportion of patients in Phenotype 1 underwent prolonged APP, with a longer duration (see Table S3). These patients were generally younger, more frequently treated in general wards, and more tolerant to prolonged proning, which may respond better to APP. Of note, these findings were based on a secondary analysis and ideally should be replicated.

This study had several strengths. First, the use of routinely available vital signs and laboratory parameters enhanced clinical applicability. Second, we validated the phenotypes in three datasets and two machine learning algorithms, and the consistency enhanced the generalizability and robustness of the findings. Third, perhaps most importantly, differential prolonged APP responses were found within the two phenotypes, offering potential insights into personalized therapies.

Some limitations must be considered. First, the absence of more biological biomarkers limited our ability to explore underlying pathophysiological mechanisms. Second, the validation cohort B used the severe national diagnosis and treatment protocol in that period, and 20.0% of patients had PaO_2_/FiO_2_ above 300 mmHg after receiving noninvasive respiratory support, which may have affected outcome assessment. However, the sensitivity analysis replicated the phenotypes after excluding these patients (Tables S12 and S13 and Figure S5). Third, our findings on prolonged APP were derived from secondary analysis, and the interaction between phenotypes and APP was limited to Day 1. Further research is necessary to confirm underlying mechanisms. Fourth, although phenotypes were stable across cohorts, additional or alternative phenotypes may emerge with additional variable inclusion. Fifth, the specific pathophysiological characteristics of COVID‐19‐related ARF may lead to more distinct phenotype separation, potentially resulting in higher than expected AUC values for predictive models. Sixth, the conclusion may only be applicable to the Chinese population. As validation cohorts were derived from COVID‐19, extensive and prospective validation of phenotype is needed in future trials.

## 4 Materials and Methods

### 4.1 Study Design and Participants

The present study was a multi‐cohort study that included three cohorts: a training cohort (non‐intubated ARF patients admitted to Chinese Database in Intensive Care of Zhongda Hospital from October 1, 2016, to October 30, 2022), a validation cohort A (non‐intubated COVID‐19‐related ARF patients from a randomized controlled trial between January 11, 2023, and April 30, 2023), and a validation cohort B (non‐intubated severe COVID‐19 patients admitted to ten hospitals in China from December 7, 2022, to January 30, 2023).

ARF patients aged 18 years or older were eligible for participation if they received non‐intubated ventilatory support on admission. Details of participating hospitals, inclusion and exclusion criteria of the cohorts were presented in the methods appendix. Ethical approval for the study protocol was obtained from the Clinical Research Ethics Committee of the affiliated Zhongda Hospital, Southeast University (Ethical Approval Number: 2022ZDSYLL177‐P01, 2023ZDSYLL022‐Y01, and 2023ZDKYSB003). This study followed the Strengthening the Reporting of Observational Studies in Epidemiology (STROBE) reporting guideline.

### 4.2 Data Collection

Details of the data collection were presented in the Supporting Information. The primary outcome was the 28‐day mortality rate in the training cohort and validation cohort B. In the validation cohort A, the primary outcome was the endotracheal intubation rate within 28 days of randomization, and the secondary outcome was mortality rate at 28 days.

### 4.3 Phenotype Derivation

Phenotypes were identified cross‐sectionally on indicators collected on Day 1. On the basis of previous research and associations with the severity or outcomes of ARF, baseline clinical data and laboratory biomarkers were considered as class‐defining variables in the model. These included BMI, PaO_2_/FiO_2_ or SpO_2_/FiO_2_ ratio, mean arterial pressure (MAP), heart rate (HR), PaCO_2_, bicarbonate, lactate, neutrophil or white blood cell count, lymphocyte count, total bilirubin, and D‐dimer. Clinical treatments and outcomes were not considered.

Data preprocessing procedures before clustering analysis involved evaluating distributions, missingness, and correlations. Variables with more than 40% missing data were removed (Table S14). Next, we applied extreme value bounding, followed by multiple imputations with chained equations (MICE) to account for missing data (Table S15). Finally, log transformation was applied to continuous variables that did not follow a normal distribution, and we excluded highly correlated variables (*r* > 0.5, Table S16 and Figure S6).

To identify and evaluate the reproducibility of the phenotypes, LPA and *k*‐means clustering were separately employed in both training and validation cohorts. For LPA, the optimal number of clusters was determined by the Akaike information criterion (AIC), Bayesian information criterion (BIC), entropy, the number of subjects assigned to each class, and the Vuong‐Lo‐Mendell‐Rubin (VLMR) test. For *k*‐means clustering, the optimal number was selected on the basis of the within‐cluster sum of squares (WCSS), gap statistic, and silhouette score. To visualize the clusters in a lower dimensional space, principal component analysis (PCA) was employed. Data processing and clustering were detailed in the additional methods. Once the optimal number was determined, continuous variables were visualized as standardized values by each cluster.

### 4.4 Statistical Analyses

Continuous variables are reported as the means (standard deviations) or medians (IQRs), whereas categorical variables are expressed as the number of cases (percentages). Differences between identified phenotypes were analyzed via Student's *t* test or Mann–Whitney *U* test for continuous variables and the chi‐square test and Fisher's exact test for categorical variables.

First, Kaplan–Meier curves were generated for time to death in all cohorts and time to intubation in the validation cohort A, and treatment differences were assessed via Cox proportional hazards models, with the results reporting as HRs and 95%CIs.

Second, in validation cohort A, the heterogeneity of the treatment effect of prolonged APP was assessed by adding the interaction term (class × prolonged APP strategy) to the models. Interactions were evaluated using Cox regressions for intubation and mortality, and logistic regression for the composite outcome.

Third, on the basis of previous research, we sought to develop a parsimonious model (with three or four variables) for predicting phenotypes. Binomial logistic regression was performed to develop the phenotype‐identification classifier. Additionally, model performance was evaluated via the AUC of the ROC curve. The results were considered statistically significant when the two‐sided *p* value was <0.05. The analysis was conducted with R version 4.4.1.

We identified two novel clinical phenotypes among non‐intubated ARF patients, with both prognostic and predictive enrichment. In validation cohort A, prolonged APP was more effective than standard care in reducing 28‐day intubation rate among patients with Phenotype 1. These findings may help tailor APP treatment to individual patient profiles.

## Author Contributions

Nan Shi contributed to the acquisition, interpretation, and drafting of the article. Ruiqiang Zheng, Xufeng Chen, Huiying Zhao, Jun Jin, Changsong Wang, Shulin Xiang, Man Huang, Hongsheng Zhao, Yi Wang, Ruixuan Yu, and Qin Sun contributed to the acquisition of data for the work. Nan Shi and Hui Chen contributed to statistical analysis. Songqiao Liu, Yi Yang, and Ling Liu contributed to funding acquisition and supervision. Jianfeng Xie, Haibo Qiu, and Hui Chen contributed to conceptualization, writing – review, editing, and supervision. All authors have read and approved the final manuscript.

## Funding

This work was supported by Noncommunicable Chronic Diseases‐National Science and Technology Major Project, Grant/Award Numbers: 2023ZD0506500; National Natural Science Foundation of China, Grant/Award Numbers: 82402565, 81930058, 82472202; Zhongda Hospital Affiliated to Southeast University, Jiangsu Province High‐Level Hospital Construction Funds, Grant/Award Number: GSP‐LCYIFH13; Nanjing Technology Development Program, Grant/Award Number: ZKX24066; National Key Research and Development Program of China, Grant/Award Number: 2022YFC2504405; and The Pilot Project of the Flagship Hospital of Integrated Traditional Chinese and Western Medicines in Zhongda Hospital Affiliated to Southeast University, Grant/Award Number: 2023zxyxt02.

## Ethics Statement

This study was conducted in accordance with the Declaration of Helsinki and approved by the Clinical Research Ethics Committee of the affiliated Zhongda Hospital, Southeast University (Ethical Approval No. 2022ZDSYLL177‐P01, 2023ZDSYLL022‐Y01, and 2023ZDKYSB003).

## Consent

Written informed consent was obtained from all participants.

## Conflicts of Interest

The authors declare no conflicts of interest.

## Supporting information




**Table S1**: Comparison of the clinical characteristics between the cohorts on Day 1.
**Table S2**: Fit statistics for latent class models from two to six in different models.
**Table S3**: Baseline characteristics, treatments, and outcomes of phenotypes using latent class analysis in the validation cohort A.
**Table S4**: Baseline characteristics, treatments, and outcomes of phenotypes using latent class analysis in the validation cohort B.
**Table S5**: Cox model of 28‐day mortality by phenotypes on Day 1.
**Table S6**: HTE results of phenotype assignment and clinical outcomes in the validation cohort A.
**Table S7**: Association between phenotype assignment and clinical outcomes in the validation cohort A.
**Table S8**: Multivariable logistic models assessing impact of variables on classification of phenotypes in the training cohort.
**Table S9**: AUC and 95%CI on classification of phenotypes in three cohorts.
**Table S10**: Multivariable logistic models assessing impact of variables on classification of phenotypes in the validation cohort A.
**Table S11**: Multivariable logistic models assessing impact of variables on classification of phenotypes in the validation cohort B.
**Table S12**: Phenotype distribution comparison of two models after excluding patients with PaO_2_/FiO_2_ above 300 mmHg in the validation cohort B.
**Table S13**: Baseline characteristics and outcomes of phenotypes using latent class analysis after excluding patients with PaO_2_/FiO_2_ above 300 mmHg in the validation cohort B.
**Table S14**: Percentage of missing data of class‐defining variables in cohorts.
**Table S15**: Class‐defining variables before and after multiple imputations on Day 1.
**Table S16**: Clinical variables for phenotyping in different datasets.
**Figure S1**: Schematic of study. ARF, acute respiratory failure; APP, awake prone positioning.
**Figure S2**: Unsupervised *k*‐means clustering in the training cohort showing the optimal number of clusters is *k* = 2.
**Figure S3**: Unsupervised *k*‐means clustering in the validation cohort A showing the optimal number of clusters is *k* = 2.
**Figure S4**: Unsupervised *k*‐means clustering in the validation cohort B showing the optimal number of clusters is *k* = 2.
**Figure S5**: Comparison of variables that contribute to clinical phenotypes in the validation cohort B after excluding patients with PaO_2_/FiO_2_ above 300 mmHg.
**Figure S6**: Heatmap of correlation between clinical variables for phenotyping.

## Data Availability

The datasets used and/or analyzed during the current study are available from the corresponding author on reasonable request.
