# Supplementary material for: Novel Phenotypes of Acute Respiratory Failure and Differential Response to Awake Prone Positioning: A Multi‐Cohort Study
Source: MedComm (2020). 2026 Jun 21;7(7):e70818. doi: 10.1002/mco2.70818 (PMC13284487; doi:10.1002/mco2.70818)
Supplement: Supplementary file 1 — Table S1: Comparison of the clinical characteristics between the cohorts on Day 1. Table S2: Fit statistics for latent class models from two to six in different models. Table S3: Baseline characteristics, treatments, and outcomes of phenotypes using latent class analysis in the validation cohort A. Table S4: Baseline characteristics, treatments, and outcomes of phenotypes using latent class analysis in the validation cohort B. Table S5: Cox model of 28‐day mortality by phenotypes on Day 1. Table S6: HTE results of phenotype assignment and clinical outcomes in the validation cohort A. Table S7: Association between phenotype assignment and clinical outcomes in the validation cohort A. Table S8: Multivariable logistic models assessing impact of variables on classification of phenotypes in the training cohort. Table S9: AUC and 95%CI on classification of phenotypes in three cohorts. Table S10: Multivariable logistic models assessing impact of variables on classification of phenotypes in the validation cohort A. Table S11: Multivariable logistic models assessing impact of variables on classification of phenotypes in the validation cohort B. Table S12: Phenotype distribution comparison of two models after excluding patients with PaO2/FiO2 above 300 mmHg in the validation cohort B. Table S13: Baseline characteristics and outcomes of phenotypes using latent class analysis after excluding patients with PaO2/FiO2 above 300 mmHg in the validation cohort B. Table S14: Percentage of missing data of class‐defining variables in cohorts. Table S15: Class‐defining variables before and after multiple imputations on Day 1. Table S16: Clinical variables for phenotyping in different datasets. Figure S1: Schematic of study. ARF, acute respiratory failure; APP, awake prone positioning. Figure S2: Unsupervised k‐means clustering in the training cohort showing the optimal number of clusters is k = 2. Figure S3: Unsupervised k‐means clustering in the validation cohort A showing the optimal n [file MCO2-7-e70818-s001.docx]

**Novel phenotypes of acute respiratory failure and differential response to awake prone positioning: a multi-cohort study**

Nan Shi^1#^, Ruiqiang Zheng^2#^, Xufeng Chen^3#^, Huiying Zhao^4^, Jun Jin^5^, Changsong Wang^6^, Shulin Xiang^7^, Man Huang^8^, Hongsheng Zhao^9^, Yi Wang^10^, Ruixuan Yu^1^, Qin Sun^1^, Hui Chen^1^, Jianfeng Xie^1^, Songqiao Liu^1,11^, Yi Yang^1^, Ling Liu^1*^, Haibo Qiu^1^; on behalf of the Chinese ARDS Research Network

^#^Nan Shi, Ruiqiang Zheng, and Xufeng Chen contributed equally to this work.

^1^Jiangsu Provincial Key Laboratory of Critical Care Medicine, Department of Critical Care Medicine, Zhongda Hospital, School of Medicine, Southeast University, Nanjing, China.

^2^Department of Critical Care Medicine, Northern Jiangsu People's Hospital, Clinical Medical School, Yangzhou University, Yangzhou, China.

^3^Department of Emergency Medicine, Emergency Department, Nanjing Medical University First Affiliated Hospital and Jiangsu Province Hospital, Nanjing, China.

^4^Department of Critical Care Medicine, Peking University People’s Hospital, Peking University, Beijing, China.

^5^Department of Critical Care Medicine, The First Affiliated Hospital of Soochow University, Soochow University, Suzhou, China.

^6^Departments of Critical Care Medicine, the First Affiliated Hospital of Harbin Medical University, Harbin, People's Republic of China.

^7^Department of Intensive Care Unit, The Peoples Hospital of Guangxi Zhuang Autonomous Region, Nanning, China.

^8^General Intensive Care Unit, Second Affiliated Hospital of Zhejiang University School of Medicine, Hangzhou, China.

^9^Department of Critical Care Medicine, Affiliated Hospital of Nantong University, Nantong, Jiangsu, China.

^10^Department of Critical Care Medicine, The First Affiliated Hospital of Xinjiang Medical University, Xinjiang Uyghur Autonomous Region, China.

^11^The First People's Hospital of Lianyungang, The Lianyungang Clinical College of Nanjing Medical University, The Affiliated Lianyungang Hospital of Xuzhou Medical University, Lianyungang , Jiangsu, China.

**Additional file**

- **Additional Methods**

1. **Inclusion and exclusion criteria**

|  | **Training cohort** | **Validation cohort A** | **Validation cohort B** |
| --- | --- | --- | --- |
| **Inclusion criteria** | 1. Age >=18-yr | 1. Age: 18-yr to 85-yr | 1. Age >=18-yr |
|  | 1. Non-intubated patients (i.e. patients using HFNC or NIV/CPAP) | 1. Confirmed COVID-19 pneumonia (with positive nucleic acid test and typical radiographic alternations) and only non-intubated patients. | 1. ICU patients with clinical manifestations consistent with COVID-19 infection or laboratory-confirmed diagnosis of COVID-19 |
|  | 1. PaO_2_/FiO_2_ <= 300 mmHg or SpO_2_/FiO_2_ <= 315† | 1. SpO_2_ <= 93% with ambient air at rest or PaO_2_/FiO_2_ <= 300 mmHg | 1. Consistent with the severe National Diagnosis and Treatment Protocol for the Novel Coronavirus (Version 10)‡ |
| **Exclusion criteria** | 1. Patients who were receiving invasive mechanical ventilation before HFNC or NIV/CPAP | 1. Definite intolerance to APP (with contraindication to proning including, but not limited to the following: pregnancy, extremity deformity, recent fracture, open thoracic or abdominal surgery, pace-maker implant in recent 48-hr, spine stability, pelvis or facial fractures, predicted difficult airway) | 1. Patients who were receiving invasive mechanical ventilation on admission |
|  | 1. Patients who died or were discharged spontaneously within 24 hours of admission | 1. Morbid obesity (BMI > 40) | 1. Patients who died or were discharged spontaneously within 24 hours of admission |
|  | 1. Duplicate records | 1. Consciousness disorder (GCS < 13), delirium, dementia | 1. Duplicate records |
|  | 1. Cardiogenic pulmonary edema | 1. Hemodynamic instability (with norepinephrine > 20 ug/min) |  |
|  |  | 1. NYHA Grade III or IV |  |
|  |  | 1. Severe hemoptysis |  |
|  |  | 1. Long term home oxygenation or CPAP |  |
|  |  | 1. Do not intubate or do not resuscitate order (DNI, DNR) |  |

Abbreviations: APP, awake prone positioning; BMI, body mass index; GCS, glasgow coma scale; NYHA, New York Heart Association; CPAP, continuous positive airway pressure; HFNC, high flow nasal cannula; NIV, non-invasive ventilation.

† When both PaO_2_/FiO_2_ and SpO_2_/FiO_2_ are available, PaO_2_/FiO_2_ should be used as the reference standard.

‡ The severe National Diagnosis and Treatment Protocol for the Novel Coronavirus (Version 10): an adult that meets any of the following criteria and cannot be explained by reasons other than COVID-19: (1) shortness of breath and respiratory rate ≥ 30 bpm; (2) SpO_2_ ≤ 93% with ambient air at rest; (3) PaO_2_/FiO_2_ ≤ 300 mmHg; (4) progressive aggravation of clinical symptoms, and lung imaging shows obvious progression of lesions within 24~48 hours > 50%.

1. **Participating hospitals and numbers of two multi-cohorts**

| Validation cohort A | Validation cohort B |
| --- | --- |
| Zhongda Hospital, Southeast University (83) | Zhongda Hospital, Southeast University (135) |
| Affiliated Hospital of Nantong University (62) | Guangxi Zhuang Autonomous Region People's Hospital (30) |
| Affiliated Hospital of Yangzhou University (50) | Harbin Medical University Affiliated Cancer Hospital (8) |
| The Affiliated Hospital of Xuzhou Medical University (20) | The Second Affiliated Hospital of Zhejiang University (25) |
| Affiliated Hangzhou First People’s Hospital, Zhejiang University School of Medicine (9) | Peking University People's Hospital (69) |
| Northern Jiangsu People's Hospital (33) | Northern Jiangsu People's Hospital (129) |
| The First Affiliated Hospital of Soochow University (28) | The First Affiliated Hospital of Nanjing Medical University (101) |
| Taizhou People's Hospital Affiliated to Nanjing Medical University (43) | The First Affiliated Hospital of Soochow University (75) |
| The First Affiliated Hospital of Xi'an Jiaotong University (11) | Affiliated Hospital of Nantong University (10) |
| The Second Affiliated Hospital of Harbin Medical University (17) | The First Affiliated Hospital of Xinjiang Medical University (27) |
| The First Hospital of Shanxi Medical University (40) |  |
| Third Hospital of Xiamen and The First Affiliated Hospital of Xiamen University (13) |  |
| All (409) | All (609) |

1. **Data collection**

In the training cohort, Day 1 was defined as the first calendar day on ICU admission. For the validation cohort A, Day 1 was defined as the first day of randomization. In the validation cohort B, Day 1 was defined as the first day of diagnosis of acute hypoxemic respiratory failure. Demographic data, clinical manifestations, chronic comorbidities, vital signs, blood gas analysis results and laboratory test results on Day 1 were extracted from electronic medical records. Treatment and outcome data were also recorded. In cases where arterial blood gas analysis was not available, PaO_2_/FiO_2_ was calculated using the Rice equation.^1^

1. **Data preprocessing**

To ensure that the data quality and hypothesis of the clustering algorithm were satisfactory, the following preprocessing steps were performed:

1. Data cleaning

Data cleaning was performed on vital monitoring and lab result data to filter out erroneous measurements and correct unsuccessful default data conversion. We then unified the original data.

1. Extreme value bounding

Because clustering algorithms are sensitive to outliers, we reserved all measurements to their mean ± 3SD to eliminate erroneous and extreme values.

1. Distribution transformation

We examined the skewness and distribution plot of the selected variables and applied log transformation to the variables when they did not conform to the normal distribution.

1. Missing value imputation

Multiple imputation by chained equations (MICE) (https://cran.rproject.org/web/packages/mice) was applied to the dataset, and all the clinical variables were modelled via random forest. MICE generated 5 independent datasets in the imputation procedure. When observations with missing variables were removed, a large number of missing values indicated a high degree of information loss. To ensure the observations contained adequate information for clustering, all patients with 40% or more missing variables were removed.

1. Correlation analysis

A correlation matrix was drawn to suppress candidate clinical variables that were too correlated (correlation coefficient > 0.5).

1. **Clustering**

We used latent profile analysis (LPA) as the clustering model in this study. Five separate models consisting of two, three, four, five and six classes were constructed, and on the basis of these five models, we subsequently determined the optimal number of clusters (k) using a combination of criteria: 1) a smaller Akaike information criterion (AIC) and Bayesian information criterion (BIC); 2) an adequate sample size within a cluster that is more than 5% of the entire study population; 3) a higher entropy, ranging from 0 to 1, for a given subject and a given cluster, where the higher the entropy, the more likely this subject is to belong to this cluster; and 4) the Vuong-Lo‒Mendell‒Rubin likelihood ratio test (which compares the fit of model k classes to k-1 classes) and clinical characteristics.^2^ Five models, comprising 2 to 6 classes, were fitted, and if one or more models had similar goodness of fit values, the model with the lowest number of classes was selected. Finally, we also selected the rank plots described above to visualize the patterns of clinical variables in the LPA.

K-means clustering analysis is a statistical methodology employed to categorize akin data points by their attributes to validate the results. The ‘‘fviz_nbclust’’ function was used to visualize the K-means cluster plot and define the number of clusters. We determined the optimal number of clusters (k) via quantitative evaluation metrics such as the within-cluster sum of squares (WCSS), gap statistic and silhouette score.^3^

WCSS measures the compactness of clusters by calculating the sum of the squared distances between the data points and their respective cluster centroids. It quantifies how close data points are to the centroids of their assigned clusters. A lower WCSS value indicates tighter, more compact clusters. The elbow method looks at the WCSS as a function of the number of clusters. One should choose a number of clusters such that adding another cluster does not substantially improve the WCSS.

Using the gap statistic method, the smallest value of k is selected as the optimal number of clusters, with the gap statistic being within one standard deviation of the gap statistic at k + 1, given k_optimal_ = min{k} and Gap(k) ≥ Gap(k+1) -𝜽_(k+1)_, where 𝜽_(k+1)_ = sd_k+1_$\sqrt{1+\frac{1}{\Omega}}$_._ The maximum gap statistic indicates the optimal estimate of the number of clusters in the data.^4^

The silhouette score measures the quality of individual data point assignments to clusters and, by extension, the overall quality of clustering. It considers both the cohesion within clusters and the separation between clusters. For each data point, the silhouette score ranges from -1 to 1, where a high score indicates that the data point is well matched to its own cluster and poorly matched to neighbouring clusters. The average silhouette score across all data points provides an overall assessment of cluster quality. A higher average silhouette score indicates better-defined clusters.

**References**

1. Rice TW, Wheeler AP, Bernard GR, et al. Comparison of the SpO2/FIO2 ratio and the PaO2/FIO2 ratio in patients with acute lung injury or ARDS. *Chest* 2007; **132**: 410-17.
2. Wilkinson SP. aphid: an R package for analysis with profile hidden Markov models. *Bioinformatics* 2019; **35**: 3829-30.
3. NbClust.pdf. Accessed August 2, 2023. https://cran.r-project.org/web/packages/NbClust/NbClust. Pdf.
4. Tibshirani R, Walther G, Hastie T. Estimating the number of clusters in a data set via the gap statistic. *Journal of the Royal Statistical Society: Series B (Statistical Methodology)* 2001; **63**: 411-23.
5. **Patients screening and inclusion of the cohorts**
6. Training cohort
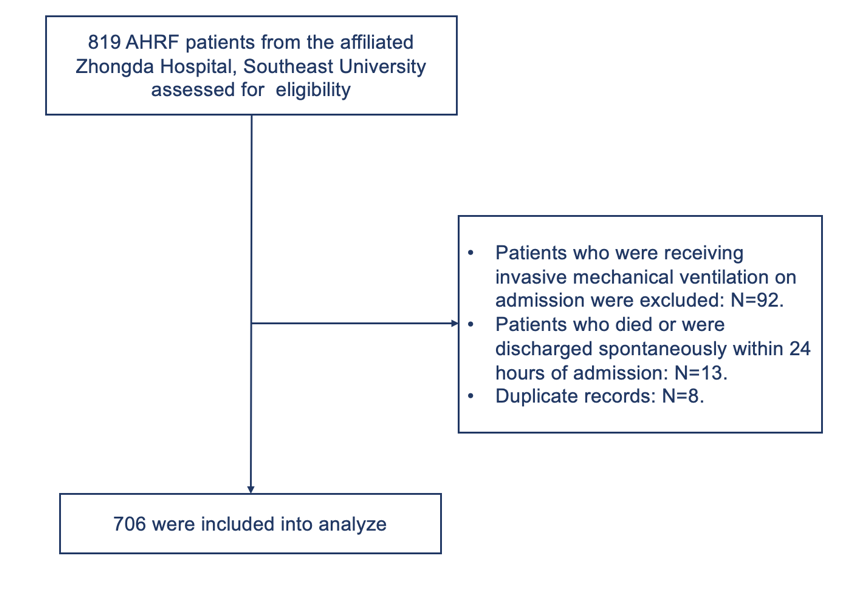

7. Validation cohort A


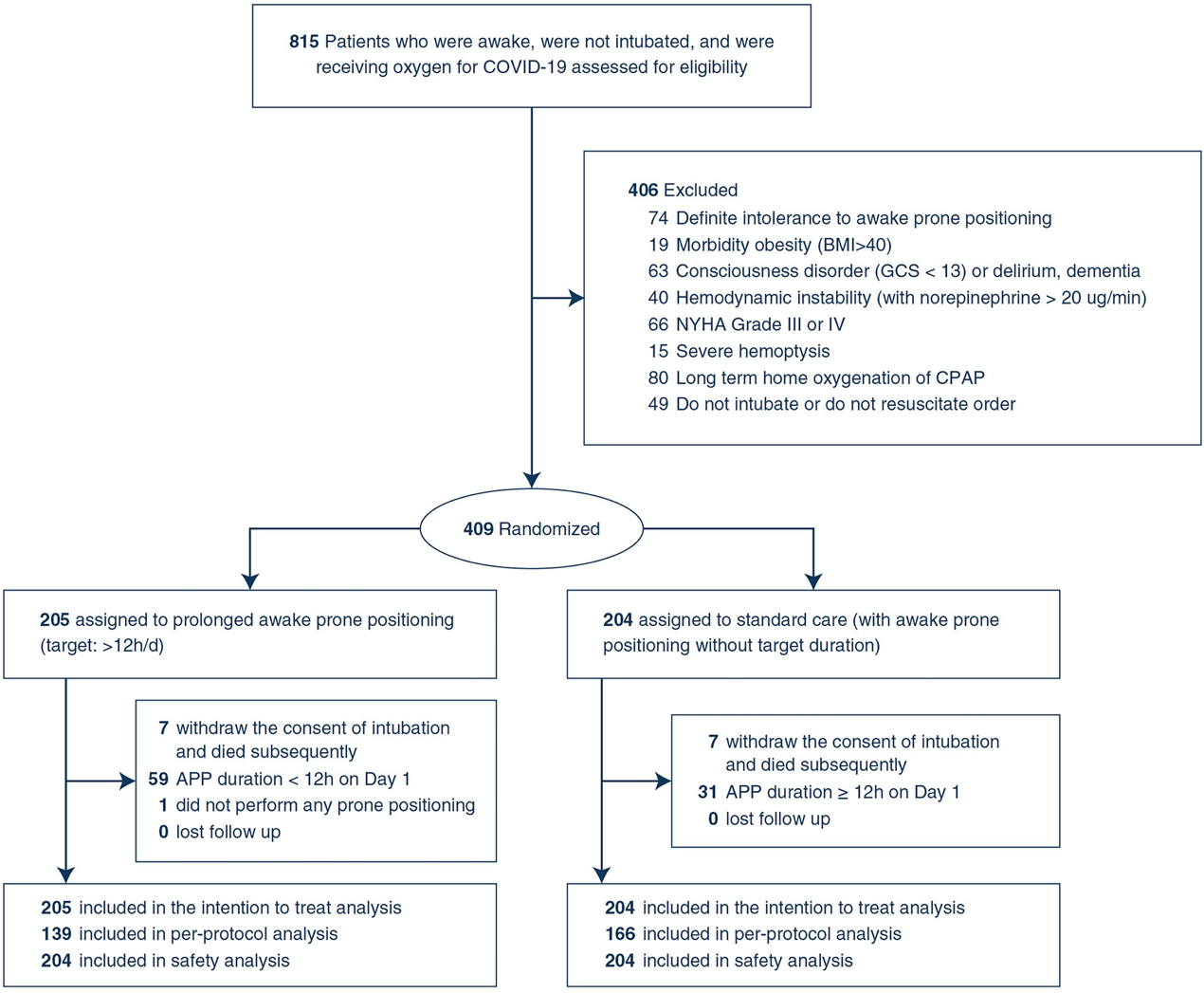


1. Validation cohort B


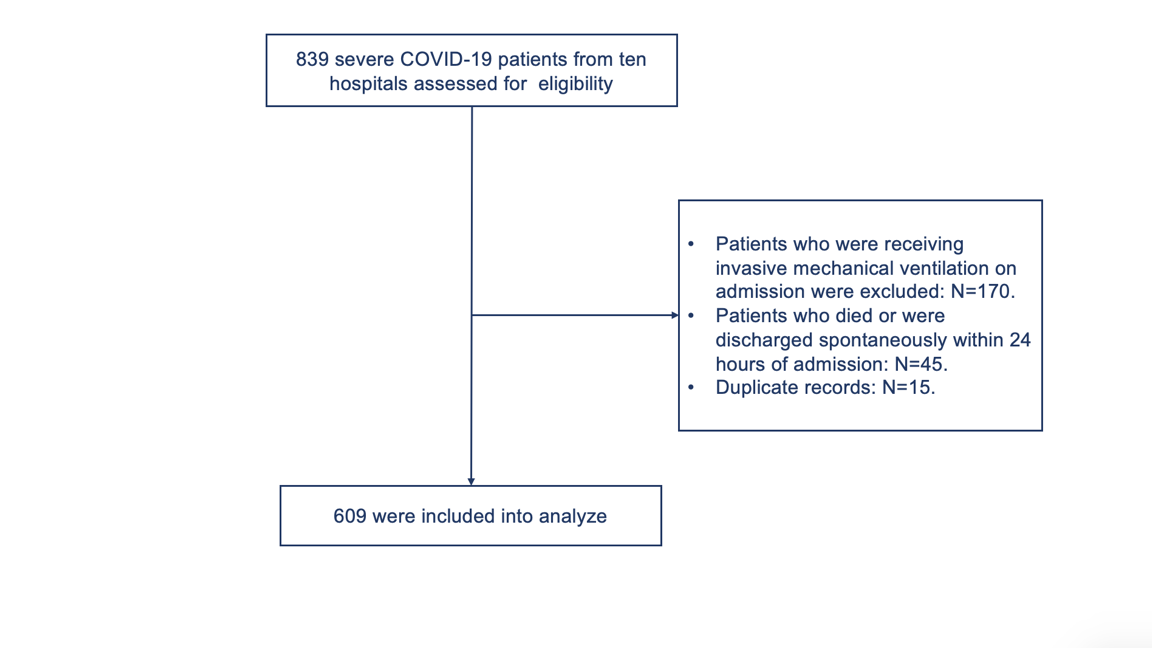


# Supplementary tables

**Supplementary Table 1: Comparison of the clinical characteristics between the cohorts on Day 1.**

| **Variables** | **Training cohort (n = 706)** | **Validation cohort A (n = 409)** | **Validation cohort B (n = 609)** | ***p* value** |
| --- | --- | --- | --- | --- |
| Age, median (IQR), years | 68 (54, 78) | 71 (64, 76) | 76 (66, 83) | < 0.001 |
| Body-mass index, median (IQR), kg/m^2^ | 23.5 (21.0, 26.1) | 24.0 (21.7, 26.0) | 23.4 (20.6, 26.0) | 0.006 |
| Sex (Male), No. (%) | 463 (65.6) | 282 (68.9) | 431 (70.8) | 0.123 |
| Coexisting illness, No. (%) |  |  |  |  |
| Chronic cardiovascular disease | 182 (25.8) | 21 (5.1) | 165 (27.1) | < 0.001 |
| Hypertension | 375 (53.1) | 214 (52.3) | 375 (61.6) | 0.002 |
| Diabetes | 192 (27.2) | 139 (34.0) | 213 (35.0) | 0.005 |
| Chronic pulmonary disease | 93 (13.2) | 10 (2.4) | 67 (11.0) | < 0.001 |
| Chronic kidney disease | 100 (14.2) | 27 (6.6) | 75 (12.3) | < 0.001 |
| Severe liver disease | 15 (2.1) | 5 (1.2) | 6 (1.0) | 0.207 |
| Others | 122 (17.3) | 37 (9.0) | 119 (19.5) | < 0.001 |
| Baseline SOFA, median (IQR) | - | 3 (2, 4) | 7 (4, 10) | < 0.001 |
| Baseline Platelet, median (IQR), ×10^9^/L | - | 180.0 (125.8, 249.3) | 159.0 (114.0, 223.0) | < 0.001 |
| Baseline Total bilirubin, median (IQR), µmol/L | 16.0 (9.5, 27.9) | 11.6 (8.4, 17.7) | 11.5 (8.2, 16.3) | < 0.001 |
| Baseline Creatinine, median (IQR), µmol/L | - | 70.0 (57.9, 97.2) | 81.0 (61.0, 135.0) | < 0.001 |
| Baseline MAP, median (IQR), mmHg | 75.2 (68.7, 85.9) | 90.0 (82.0, 98.0) | 89.3 (79.3, 99.3) | < 0.001 |
| HR, median (IQR), bpm | 100 (97, 102) | 84 (75, 95) | 94 (80, 108) | < 0.001 |
| Lymphocyte count, median (IQR), ×10^9^/L | 0.70 (0.44, 1.02) | 0.71 (0.50, 1.08) | 0.54 (0.35, 0.81) | < 0.001 |
| D-dimer, median (IQR), µg/mL | 1.8 (0.8, 3.1) | 1.5 (0.7, 4.5) | 2.2 (1.0, 6.0) | < 0.001 |
| PaO_2_, median (IQR), mmHg | 75.0 (62.0, 88.6) | - | 90.6 (72.0, 121.8) | < 0.001 |
| FiO_2_, median (IQR), % | 41 (38, 50) | 41 (33, 50) | 50 (40, 61) | < 0.001 |
| PaCO_2_, median (IQR), mmHg | 41.6 (35.8, 47.8) | 35.0 (31.4, 40.9) | 35.0 (30.0, 42.0) | < 0.001 |
| Bicarbonate, median (IQR), mmol/L | 21.6 (18.6, 24.3) | 23.6 (21.3, 26.4) | 22.4 (19.5, 26.3) | < 0.001 |
| Lactate, median (IQR), mmol/L | 2.0 (1.5, 2.7) | 1.8 (1.3, 2.6) | 1.7 (1.2, 2.4) | < 0.001 |

Abbreviations: SOFA, sequential organ failure assessment; MAP, mean arterial pressure; HR, heart rate.

**Supplementary Table 2: Fit statistics for latent class models from two to six in different models.**

**(A) the training cohort on Day 1**

| **k** | **AIC** | **BIC** | **Entropy†** | **Number of individuals** | | ***p* value**  **k vs k-1**  **classes‡** |
| --- | --- | --- | --- | --- | --- | --- |
|  |  |  |  | **Minimum** | **Maximum** |  |
| 2 Classes | 19916 | 19959 | 0.698 | 127 | 579 | 0.01 |
| 3 Classes | 19867 | 19925 | 0.639 | 131 | 430 | 0.01 |
| 4 Classes | 19771 | 19845 | 0.671 | 28 | 399 | 0.01 |
| 5 Classes | 19712 | 19801 | 0.655 | 34 | 349 | 0.01 |
| 5 Classes | 19702 | 19806 | 0.643 | 31 | 317 | 0.03 |

**(B) the validation cohort A on Day 1**

| **k** | **AIC** | **BIC** | **Entropy†** | **Number of individuals** | | ***p* value**  **k vs k-1**  **classes‡** |
| --- | --- | --- | --- | --- | --- | --- |
|  |  |  |  | **Minimum** | **Maximum** |  |
| 2 Classes | 11505 | 11531 | 0.647 | 127 | 282 | 0.01 |
| 3 Classes | 11422 | 11457 | 0.785 | 12 | 277 | 0.01 |
| 4 Classes | 11392 | 11436 | 0.691 | 12 | 206 | 0.01 |
| 5 Classes | 11387 | 11441 | 0.733 | 6 | 219 | 0.04 |
| 5 Classes | 11386 | 11449 | 0.654 | 7 | 123 | 0.129 |

**(C) the validation cohort B on Day 1**

| **k** | **AIC** | **BIC** | **Entropy†** | **Number of individuals** | | ***p* value**  **k vs k-1**  **classes‡** |
| --- | --- | --- | --- | --- | --- | --- |
|  |  |  |  | **Minimum** | **Maximum** |  |
| 2 Classes | 17182 | 17221 | 0.576 | 211 | 398 | 0.01 |
| 3 Classes | 17130 | 17182 | 0.617 | 111 | 358 | 0.01 |
| 4 Classes | 17114 | 17179 | 0.642 | 52 | 320 | 0.01 |
| 5 Classes | 17070 | 17149 | 0.656 | 38 | 306 | 0.01 |
| 5 Classes | 17047 | 17140 | 0.706 | 31 | 304 | 0.01 |

†Entropy is an index of how well the classes are separated: it ranges from zero to one and values of about 0·8 or higher are thought to be a sign of a useful mode

‡p value represents the Vuong-Lo-Mendell-Rubin (VLMR) test, which test if k classes is a better model fit than k-1 classes.

Abbreviations: AIC, Akaike Information Criterion; BIC, Bayesian Information Criterion.

**Supplementary Table 3: Baseline characteristics, treatments and outcomes of phenotypes using latent class analysis in the validation cohort A.**

|  | **All**  **(n=409)** | **Phenotype 1**  **(n=282)** | **Phenotype 2**  **(n=127)** | | ***p* value** |  |  |
| --- | --- | --- | --- | --- | --- | --- | --- |
| Age, median (IQR), years | 71 (64,76) | 70 (62,75) | 73 (67,77) | | < 0.001 |  |  |
| Sex (Male), No (%) | 282 (68.9) | 199 (70.6) | 83 (65.4) | | 0.348 |  |  |
| Body-mass index, median (IQR), kg/m^2^ | 24.0 (21.7,26.0) | 23.9 (21.7,25.9) | 24.2 (22.0,26.4) | | 0.830 |  |  |
| Time from hospital admission to randomization, median (IQR), d | 1 (0,2) | 1 (0,2) | 1 (0,3) | | 0.703 |  |  |
| Oxygenation mode, No. (%) |  |  |  | | < 0.001 |  |  |
| Standard oxygenation | 263 (64.3) | 215 (76.2) | 48 (37.8) | |  |  |  |
| High-flow nasal oxygen | 109 (26.7) | 53 (18.8) | 56 (44.1) | |  |  |  |
| Mask noninvasive ventilation | 37 (9.0) | 14 (5.0) | 23 (18.1) | |  |  |  |
| SOFA score, median (IQR) | 3 (2,4) | 3 (2,4) | 4 (3,6) | | < 0.001 |  |  |
| Location at enrollment, No. (%) |  |  |  | | < 0.001 |  |  |
| Intensive care unit | 37 (9.0) | 16 (5.7) | 21 (16.5) | |  |  |  |
| Intermediate care unit | 105 (25.7) | 52 (18.4) | 53 (41.7) | |  |  |  |
| General ward | 267 (65.3) | 214 (75.9) | 53 (41.7) | |  |  |  |
| **Coexisting illness, No. (%)** |  |  |  | |  |  |  |
| Chronic heart disease | 21 (5.1) | 12 (4.3) | 9 (7.1) | | 0.338 |  |  |
| Hypertension | 214 (52.3) | 143 (50.7) | 71 (55.9) | | 0.386 |  |  |
| Diabetes | 139 (34.0) | 84 (29.8) | 55 (43.3) | | 0.011 |  |  |
| Chronic kidney disease | 27 (6.6) | 14 (5.0) | 13 (10.2) | | 0.076 |  |  |
| Chronic pulmonary disease | 10 (2.4) | 8 (2.8) | 2 (1.6) | | 0.731 |  |  |
| Severe liver disease | 5 (1.2) | 5 (1.8) | 0 | | 0.330 |  |  |
| Others | 37 (9.0) | 26 (9.2) | 11 (8.7) | | 1.000 |  |  |
| **Vital signs and labs** |  |  | |  |  | |  |
| Heart rate, median (IQR),bpm | 84 (75, 95) | 82 (74, 90) | 90 (80, 101) | | < 0.001 |  |  |
| Mean arterial pressure, mean (SD), mmHg | 89.0 (82.0, 98.5) | 89.0 (80.0, 98.0) | 92.0 (84.0, 101.5) | | 0.007 | |  |
| Platelet, median (IQR), ×10^9^/L | 180 (126, 249) | 181 (128, 251) | 179 (123, 243) | | 0.491 |  |  |
| Total bilirubin, median (IQR),µmol/L | 11.6 (8.4, 17.7) | 11.4 (8.2, 16.3) | 13.3 (8.7, 21.7) | | 0.011 |  |  |
| Creatinine, median (IQR),µmol/L | 70.0 (57.9, 97.2) | 69.0 (57.3, 88.0) | 74.4 (58.0, 145.0) | | 0.029 | | |
| SpO_2_/FiO_2_ ratio, median (IQR) | 234.2 (190.0, 290.9) | 255.8 (222.0, 297.0) | 180.0 (156.1, 231.7) | | < 0.001 |  |  |
| FiO_2_, median (IQR), % | 41 (33, 50) | 37 (33, 41) | 50 (41, 60) | | < 0.001 |  |  |
| White blood cell count, median (IQR),×10^9^/L | 7.7 (5.3, 10.9) | 6.5 (4.9, 9.4) | 10.5 (7.7, 13.4) | | < 0.001 |  |  |
| Lymphocyte count, median (IQR), ×10^9^/L | 0.70 (0.49, 1.12) | 0.79 (0.55, 1.26) | 0.60 (0.34, 0.81) | | < 0.001 |  |  |
| D-dimer, median (IQR),µg/mL | 1.6 (0.8, 4.8) | 1.0 (0.6, 1.8) | 6.6 (3.6, 15.2) | | < 0.001 |  |  |
| PaCO_2_, median (IQR),mmHg | 35.0 (31.1, 40.9) | 36.0 (32.0, 40.9) | 33.5 (30.0, 40.8) | | 0.007 |  |  |
| Bicarbonate, median (IQR),mmol/L | 23.9 (21.4, 26.5) | 24.3 (21.9, 26.5) | 23.1 (19.8, 26.2) | | 0.006 |  |  |
| Lactate, median (IQR),mmol/L | 1.8 (1.3, 2.7) | 1.6 (1.2, 2.3) | 2.1 (1.6, 3.4) | | < 0.001 |  |  |

Continued

|  | **All**  **(n=409)** | **Phenotype 1**  **(n=282)** | **Phenotype 2**  **(n=127)** | ***p* value** |
| --- | --- | --- | --- | --- |
| **Treatment and outcomes** |  |  |  |  |
| Pharmacological intervention, No. (%) |  |  |  |  |
| Glucocorticoids for treatment of COVID-19 | 254 (62.3) | 165 (58.7) | 89 (70.1) | 0.037 |
| Antiviral drugs | 257 (62.8) | 163 (57.8) | 94 (74.0) | 0.014 |
| Anticoagulants | 343 (84.1) | 231 (82.2) | 112 (88.2) | 0.167 |
| Prolonged awake prone positioning, n (%) | 205 (50.1) | 152 (53.9) | 53 (41.7) | 0.030 |
| The duration of prolonged awake prone positioning, hour | |  |  |  |
| Day 1 | 10.0 (4.0, 12.5) | 10.5 (4.0, 13.0) | 8.0 (4.0, 12.0) | 0.040 |
| Day 2 | 10.0 (5.0, 13.0) | 11.0 (5.0, 13.0) | 9.0 (4.5, 12.0) | 0.094 |
| Day 3 | 11.0 (4.0, 13.0) | 12.0 (5.0, 13.0) | 10.0 (4.0, 13.0) | 0.227 |
| Day 4 | 10.0 (4.0, 13.0) | 11.0 (4.0, 13.0) | 10.0 (4.0, 13.0) | 0.311 |
| Day 5 | 11.3 (4.1, 13.0) | 12.0 (5.0, 13.0) | 10.0 (3.3, 13.0) | 0.146 |
| Day 6 | 11.0 (4.3, 13.0) | 12.0 (5.0, 13.0) | 10.0 (4.0, 13.0) | 0.411 |
| Day 7 | 11.0 (4.0, 13.0) | 11.0 (5.0, 13.0) | 9.0 (3.0, 13.0) | 0.152 |
| Intubation within 28 days of randomization, No. (%) | 91 (22.2) | 27 (9.6) | 64 (50.4) | < 0.001 |
| Mortality at 28 days, No. (%) | 93 (22.7) | 30 (10.6) | 63 (49.6) | < 0.001 |
| Intubation or death at 28 days, No. (%) | 105 (25.7) | 37 (13.1) | 68 (53.5) | < 0.001 |
| Hospital LOS, day | 13 (8,19) | 13 (8, 18) | 14 (8, 21) | 0.387 |

Abbreviations: SOFA, sequential organ failure assessment; LOS, length of stay.

**Supplementary Table 4: Baseline characteristics, treatments and outcomes of phenotypes using latent class analysis in the validation cohort B.**

|  | **All**  **(n=609)** | **Phenotype 1**  **(n = 398)** | **Phenotype 2**  **(n = 211)** | ***p* value** |
| --- | --- | --- | --- | --- |
| Age, median (IQR), years | 76 (66, 83) | 75 (66, 83) | 76 (67, 84) | 0.826 |
| Sex (Male), No (%) | 431 (70.8) | 272 (68.3) | 159 (75.4) | 0.086 |
| Body-mass index, mean (SD) , kg/m^2^ | 23.4 (4.3) | 23.8 (4.5) | 22.5 (3.8) | < 0.001 |
| SOFA, median (IQR) | 7 (4, 10) | 6 (4, 9) | 9 (6, 12) | < 0.001 |
| Fever day before admission, median (IQR), day | 3 (0, 8) | 2 (0, 7) | 5 (0, 10) | 0.031 |
| Maximum temperature before admission, median (IQR), ℃ | 38.6 (38.1, 39.0) | 38.6 (38.1, 39.0) | 38.6 (38.1, 39.0) | 0.258 |
| **Symptoms, No%** |  |  |  |  |
| Cough | 415 (68.1) | 252 (63.3) | 163 (77.3) | < 0.001 |
| Weak | 182 (29.9) | 121 (30.4) | 61 (28.9) | 0.772 |
| Hyposmia | 9 (1.5) | 9 (2.3) | 0 (0) | 0.031 |
| Hypogeusia | 11 (1.8) | 10 (2.5) | 1 (0.5) | 0.108 |
| Rhinobyon | 14 (2.3) | 9 (2.3) | 5 (2.3) | 1 |
| Sore throat | 37 (6.1) | 24 (6.0) | 13 (6.2) | 1 |
| Diarrhea | 14 (2.3) | 7 (1.8) | 7 (3.3) | 0.259 |
| Conjunctivitis | 3 (0.5) | 2 (0.5) | 1 (0.5) | 1 |
| Myalgia | 55 (9.0) | 41 (10.3) | 14 (6.6) | 0.176 |
| **Coexisting illness, No. (%)** |  |  |  |  |
| Chronic heart disease, | 165 (27.1) | 106 (26.6) | 59 (28.0) | 0.798 |
| Hypertension | 375 (61.6) | 252 (63.3) | 123 (58.3) | 0.261 |
| Diabetes | 213 (35.0) | 145 (36.4) | 68 (32.2) | 0.344 |
| Chronic kidney disease | 75 (12.3) | 45 (11.3) | 30 (14.2) | 0.362 |
| Cirrhosis | 6 (1.0) | 2 (0.5) | 4 (1.9) | 0.189 |
| Chronic pulmonary disease | 67 (11.0) | 48 (12.1) | 19 (9.0) | 0.312 |
| Others | 119 (19.5) | 80 (20.1) | 39 (18.5) | 0.710 |
| **Vital signs** |  |  |  |  |
| Heart rate, median (IQR),bpm | 94 (80, 109) | 90 (77, 102) | 100 (87, 115) | < 0.001 |
| Respiratory rate, median (IQR),bpm | 22 (18, 28) | 22 (18, 28) | 23 (19, 30) | 0.213 |
| Mean arterial pressure, mean (SD), mmHg | 89.7 (15.7) | 90.1 (15.4) | 89.0 (16.3) | 0.477 |
| **Laboratory results** |  |  |  |  |
| PaO_2_/FiO_2_ ratio, median (IQR), mmHg | 179.3 (124.3, 267.7) | 215.9 (145.7, 308.1) | 130.4 (94.8, 186.6) | < 0.001 |
| FiO_2_, median (IQR), % | 50 (40, 61) | 45 (40, 60) | 60 (50, 80) | < 0.001 |
| Total bilirubin, median (IQR),µmol/L | 11.7 (8.2, 16.3) | 10.8 (7.5, 14.5) | 13.3 (10.0, 19.5) | < 0.001 |
| Albumin, median (IQR), g/L | 29.7 (27.2, 33.3) | 29.9 (27.2, 34.2) | 29.3 (27.3, 32.0) | 0.176 |
| ALT, median (IQR), U/L | 26.0 (16.8, 45.0) | 25.7 (16.0, 41.0) | 27.0 (18.8, 52.5) | 0.053 |
| AST, median (IQR), U/L | 39.0 (24.5, 57.0) | 35.0 (24.0, 53.0) | 43.5 (26.0, 64.2) | 0.007 |
| LDH, median (IQR), U/L | 372.1 (267.2, 510.5) | 350.5 (252.5, 448.2) | 446.0 (308.6, 605.3) | < 0.001 |
| Creatinine, median (IQR),µmol/L | 82.3 (61.0, 136.3) | 79.0 (61.0, 129.0) | 97.9 (63.5, 156.0) | 0.064 |
| BUN, median (IQR), μmol/L | 9.4 (6.4, 15.5) | 8.7 (5.9, 14.3) | 10.2 (7.2, 17.6) | 0.004 |
| CK, median (IQR), U/L | 110.0 (45.9, 265.7) | 105.0 (47.0, 250.0) | 111.0 (44.9, 297.5) | 0.582 |

Continued

|  | **All**  **(n=609)** | **Phenotype 1**  **(n = 398)** | **Phenotype 2**  **(n = 211)** | ***p* value** |
| --- | --- | --- | --- | --- |
| Neutrophils, median (IQR),×10^9^/L | 8.0 (5.1, 11.7) | 6.7 (4.5, 9.8) | 10.7 (7.8,15.4) | < 0.001 |
| Lymphocytes, median (IQR), ×10^9^/L | 0.55 (0.35, 0.81) | 0.58 (0.36, 0.87) | 0.51 (0.35, 0.71) | 0.040 |
| CRP, median (IQR), mg/L | 79.7 (36.8, 132.8) | 67.8 (24.1, 115.4) | 90.0 (53.0, 176.4) | < 0.001 |
| IL-6, median (IQR), pg/mL | 53.3 (13.2, 180.7) | 42.1 (10.4, 149.6) | 90.5 (28.3, 298.0) | 0.009 |
| Platelet, median (IQR), ×10^9^/L | 160 (115, 222) | 163 (114, 223) | 158 (116, 220) | 0.934 |
| D-dimer, median (IQR), µg/mL | 2.2 (1.1, 5.9) | 1.4 (0.8, 2.3) | 10.1 (5.2, 16.7) | < 0.001 |
| PaCO_2_, median (IQR),mmHg | 35.0 (30.0, 42.1) | 35.2 (30.0, 42.1) | 34.8 (29.5, 42.1) | 0.397 |
| Bicarbonate, median (IQR),mmol/L | 22.2 (19.1, 25.5) | 23.0 (19.6, 26.8) | 21.5 (18.4, 24.5) | 0.004 |
| Lactate, median (IQR),mmol/L | 1.7 (1.2, 2.4) | 1.5 (1.1, 2.0) | 2.3 (1.6, 3.6) | < 0.001 |
| TnI, median (IQR), ng/mL | 0.03 (0.01, 0.10) | 0.03 (0.01, 0.07) | 0.05 (0.01, 0.16) | 0.006 |
| CKMB, median (IQR), U/L | 3.7 (2.0, 9.3) | 3.6 (2.0, 8.3) | 3.8 (2.0, 9.8) | 0.460 |
| PT, median (IQR), s | 14.3 (12.6, 28.1) | 14.1 (12.4, 29.3) | 15.6 (13.0, 24.6) | 0.445 |
| APTT, median (IQR), s | 31.1 (26.9, 35.7) | 31.1 (27.3, 35.6) | 31.1 (26.5, 36.3) | 0.947 |
| HFNO | 248 (40.7) | 171 (43.0) | 77 (36.5) | 0.144 |
| NIV | 260 (42.7) | 180 (45.2) | 80 (37.9) | 0.099 |
| **Pharmacological intervention, No. (%)** |  |  |  |  |
| Antiviral drugs | 400 (65.7) | 260 (65.3) | 140 (66.4) | 0.870 |
| Glucocorticoids for treatment of COVID-19 | 406 (66.7) | 249 (62.6) | 157 (74.4) | 0.004 |
| Anticoagulants | 445 (73.1) | 280 (70.4) | 165 (78.2) | 0.048 |
| Thymosin | 238 (39.1) | 139 (34.9) | 99 (46.9) | 0.005 |
| Vasopressor | 339 (55.7) | 198 (49.7) | 141 (66.8) | < 0.001 |
| **Other intervention, No. (%)** |  |  |  |  |
| CRRT | 107 (17.6) | 62 (15.6) | 45 (21.3) | 0.096 |
| Prone positioning | 288 (47.3) | 177 (44.5) | 111 (52.6) | 0.068 |
| ECMO | 9 (1.5) | 3 (0.8) | 6 (2.8) | 0.071 |
| AKI, No. (%) | 188 (30.9) | 104 (26.1) | 84 (39.8) | < 0.001 |
| Intubation at 28 days, No. (%) | 345 (56.7) | 198 (49.7) | 147 (69.7) | < 0.001 |
| 28-day in-hospital mortality, No. (%) | 230 (37.8) | 125 (31.4) | 105 (49.8) | < 0.001 |
| Intubation or death at 28 days, No. (%) | 406 (66.7) | 239 (60.1) | 167 (79.1) | < 0.001 |
| Hospital LOS, median (IQR), mmHg | 16 (9, 26) | 15 (9, 23) | 16 (9, 28) | 0.246 |

Abbreviations: SOFA, sequential organ failure assessment; bpm, beats per minute; ALT, alanine aminotransferase; AST, aspartate aminotransferase; LDH, lactate dehydrogenase; BUN, blood urea nitrogen; CK, creatine kinase; CRP, C-reaction protein; IL-6, interleukin-6; TnI, troponin I; CKMB, creatine kinase lsoenzyme; PT, prothrombin time; APTT, activated partial thromboplastin time; HFNO, high flow nasal oxygen; NIV, non-invasive ventilation; CRRT, continuous renal replacement therapy; ECMO, extracorporeal membrane oxygenation; AKI, acute kidney injury; LOS, length of stay.

**Supplementary Table 5: Cox model of 28-day mortality by phenotypes on Day 1.**

|  |  | **Hazard ratio** | **95% CI** |
| --- | --- | --- | --- |
| The training cohort | Phenotype 2 (vs 1) | 2.48 | (1.57, 3.90) |
| The validation cohort A | Phenotype 2 (vs 1) | 6.06 | (3.92, 9.38) |
| The validation cohort B | Phenotype 2 (vs 1) | 1.83 | (1.41, 2.38) |

Concordance index, 0.584 in the training cohort, 0.711 in the validation cohort A and 0.572 in the validation cohort B.

**Supplementary Table 6: HTE results of phenotype assignment and clinical outcomes in the validation cohort A.**

| **Outcomes** | **Phenotype 1 (n = 282)** | | **Phenotype 2 (n = 127)** | |
| --- | --- | --- | --- | --- |
|  | **Prolonged APP**  **(n = 152)** | **Standard care**  **(n = 130)** | **Prolonged APP**  **(n = 53)** | **Standard care**  **(n = 74)** |
| Intubation within 28 days of  randomization, No. (%) | HR 0.35, 95%CI (0.14, 0.93), *P* = 0.035 | | HR 0.38, 95%CI (0.14, 0.99), Adjusted *P* = 0.048 | |
| Mortality at 28 days, No. (%) | HR 0.49, 95%CI (0.20, 1.19), *P* = 0.114 | | HR 0.52, 95%CI (0.21, 1.29), Adjusted *P* = 0.160 | |
| Intubation or death at 28 days, No. (%) | OR 0.38, 95%CI (0.14, 1.05), *P* = 0.061 | | OR 0.41, 95%CI (0.15, 1.15), Adjusted *P* = 0.090 | |

HTE, heterogeneity of the treatment effect; Adjusted for age, sex, and baseline SpO_2_/FiO_2_.

Compared to phenotype 2, Phenotype 1

**Supplementary Table 7: Association between phenotype assignment and clinical outcomes in the validation cohort A.**

| **Outcomes** | **Phenotype 1 (n = 282)** | | | **Phenotype 2 (n = 127)** | | |
| --- | --- | --- | --- | --- | --- | --- |
|  | **Prolonged APP (n = 152)** | **Standard care (n = 130)** | ***p* value** | **Prolonged APP (n = 53)** | **Standard care (n = 74)** | ***p* value** |
| Intubation within 28 days of  randomization, No. (%) | 8 (5.3) | 19 (14.6) | 0.014 | 27 (50.9) | 37 (50.0) | 1.000 |
| Mortality at 28 days, No. (%) | 11 (7.2) | 19 (14.6) | 0.070 | 27 (50.9) | 36 (48.6) | 0.940 |
| Intubation or death at 28 days, No. (%) | 13 (8.6) | 24 (18.5) | 0.023 | 29 (54.7) | 39 (52.7) | 0.965 |

**Supplementary Table 8: Multivariable logistic models assessing impact of variables on classification of phenotypes in the training cohort.**

|  | Three-variable model | | *p* value | Four-variable model | | *p* value |
| --- | --- | --- | --- | --- | --- | --- |
|  | OR | 95% CI |  | OR | 95% CI |  |
| PaO_2_/FiO_2_ ratio | 0.99 | (0.98, 0.99) | < 0.001 | 0.99 | (0.98, 0.99) | < 0.001 |
| White blood cell | 1.10 | (1.07, 1.13) | < 0.001 | 1.11 | (1.06, 1.17) | < 0.001 |
| D-dimer | 1.00 | (1.00, 1.00) | < 0.001 | 1.00 | (1.00, 1.00) | < 0.001 |
| Lactate | - | - | - | 15.0 | (9.14, 26.9) | < 0.001 |

Compared to phenotype 1, Phenotype 2

**Supplementary Table 9: AUC and 95%CI on classification of phenotypes in three cohorts.**

|  | Three-variable model | | Four-variable model | |
| --- | --- | --- | --- | --- |
|  | AUC | 95% CI | AUC | 95% CI |
| The training cohort | 0.774 | (0.727, 0.821) | 0.976 | (0.965, 0.986) |
| The validation cohort A | 0.964 | (0.948, 0.979) | 0.974 | (0.962, 0.987) |
| The validation cohort B | 0.981 | (0.972, 0.990) | 0.986 | (0.979, 0.993) |

Compared to phenotype 1, Phenotype 2

**Supplementary Table 10: Multivariable logistic models assessing impact of variables on classification of phenotypes in the validation cohort A.**

|  | Three-variable model | | *p* value | Four-variable model | | *p* value |
| --- | --- | --- | --- | --- | --- | --- |
|  | OR | 95% CI |  | OR | 95% CI |  |
| SpO_2_/FiO_2_ ratio | 0.98 | (0.97, 0.98) | < 0.001 | 0.97 | (0.96, 0.98) | < 0.001 |
| White blood cell | 1.35 | (1.22, 1.50) | < 0.001 | 1.34 | (1.20, 1.52) | < 0.001 |
| D-dimer | 1.96 | (1.67, 2.37) | < 0.001 | 2.01 | (1.69, 2.47) | < 0.001 |
| Lactate | - | - | - | 2.11 | (1.53, 3.01) | < 0.001 |

Compared to phenotype 1, Phenotype 2

**Supplementary Table 11: Multivariable logistic models assessing impact of variables on classification of phenotypes in the validation cohort B.**

|  | Three-variable model | | *p* value | Four-variable model | | *p* value |
| --- | --- | --- | --- | --- | --- | --- |
|  | OR | 95% CI |  | OR | 95% CI |  |
| PaO_2_/FiO_2_ ratio | 0.99 | (0.98, 0.99) | < 0.001 | 0.98 | (0.98, 0.99) | < 0.001 |
| Neutrophil | 1.28 | (1.19, 1.39) | < 0.001 | 1.29 | (1.19, 1.41) | < 0.001 |
| D-dimer | 1.00 | (1.00, 1.00) | < 0.001 | 1.00 | (1.00, 1.00) | < 0.001 |
| Lactate | - | - | - | 1.59 | (1.34, 1.93) | < 0.001 |

Compared to phenotype 1, Phenotype 2

**Supplementary Table 12: Phenotype distribution comparison of two models after excluding patients with PaO_2_/FiO_2_ above 300 mmHg in the validation cohort B.**

|  | Phenotype 1 (New) | Phenotype 2 (New) |
| --- | --- | --- |
| Phenotype 1 (Original) | 290 | 0 |
| Phenotype 2 (Original) | 30 | 167 |

**Supplementary Table 13: Baseline characteristics, and outcomes of phenotypes using latent class analysis after excluding patients with PaO_2_/FiO_2_ above 300 mmHg in the validation cohort B.**

|  | **All**  **(n=487)** | **Phenotype 1**  **(n = 320)** | **Phenotype 2**  **(n = 167)** | ***p* value** |
| --- | --- | --- | --- | --- |
| Age, median (IQR), years | 76 (67, 83) | 76 (67, 83) | 76 (68, 84) | 0.830 |
| Sex (Male), No (%) | 354 (72.7) | 225 (70.3) | 129 (77.3) | 0.128 |
| Body-mass index, mean (SD) , kg/m^2^ | 23.1 (4.1) | 23.3 ± 4.3 | 22.6 ± 3.7 | 0.051 |
| SOFA, median (IQR) | 7 (4, 10) | 7 (4, 10) | 7 (5, 11) | 0.140 |
| **Laboratory results** |  |  |  |  |
| PaO_2_/FiO_2_ ratio, median (IQR), mmHg | 154.0 (112.6, 212.1) | 170.3 (126.0, 229.3) | 127.0 (89.6, 168.6) | < 0.001 |
| FiO_2_, median (IQR), % | 50 (40, 70) | 50 (40, 60) | 61 (50, 80) | < 0.001 |
| Total bilirubin, median (IQR),µmol/L | 12.4 (8.7, 17.1) | 11.3 (8.2, 15.9) | 14.5 (10.1, 19.1) | < 0.001 |
| Albumin, median (IQR), g/L | 29.5 (26.9, 32.8) | 29.5 (26.8, 33.5) | 29.4 (27.1, 32.0) | 0.381 |
| ALT, median (IQR), U/L | 25.0 (16.0, 42.1) | 25.0 (16.0, 41.0) | 25.0 (16.0, 45.0) | 0.269 |
| AST, median (IQR), U/L | 40.0 (25.6, 59.5) | 36.0 (24.0, 54.8) | 43.0 (29.1, 70.7) | 0.003 |
| LDH, median (IQR), U/L | 372.1 (274.0, 521.0) | 350.0 (257.3, 454.0) | 458.0 (305.5, 621.5) | < 0.001 |
| Creatinine, median (IQR),µmol/L | 84.5 (63.2, 137.5) | 80.5 (62.0, 127.7) | 101.0 (67.0, 156.0) | 0.026 |
| BUN, median (IQR), μmol/L | 9.4 (6.5, 15.5) | 9.0 (5.9, 14.4) | 10.2 (7.0, 17.6) | 0.003 |
| CK, median (IQR), U/L | 127.0 (50.3, 287.0) | 118.7 (49.0, 271.0) | 140.0 (58.7, 302.0) | 0.237 |
| Neutrophils, median (IQR),×10^9^/L | 8.27 (5.44, 11.88) | 6.97 (4.72, 10.25) | 10.39 (7.71, 15.53) | < 0.001 |
| Lymphocytes, median (IQR), ×10^9^/L | 0.54 (0.35, 0.80) | 0.58 (0.35, 0.85) | 0.46 (0.35, 0.70) | 0.040 |
| CRP, median (IQR), mg/L | 82.5 (46.2, 140.6) | 79.1 (39.3, 130.9) | 90.0 (53.5, 161.8) | 0.010 |
| Platelet, median (IQR), ×10^9^/L | 164 (112, 222) | 158 (109, 221) | 176 (125, 222) | 0.174 |
| D-dimer, median (IQR), µg/mL | 2.3 (1.1, 7.3) | 1.4 (0.9, 2.3) | 11.3 (6.4, 19.9) | < 0.001 |
| PaCO_2_, median (IQR),mmHg | 34.9 (30.0, 42.0) | 35.0 (30.0, 42.0) | 34.8 (30.0, 42.1) | 0.962 |
| Bicarbonate, median (IQR),mmol/L | 22.4 (19.5, 26.4) | 22.7 (19.5, 27.2) | 21.9 (19.5, 25.3) | 0.049 |
| Lactate, median (IQR),mmol/L | 1.7 (1.2, 2.4) | 1.6 (1.1, 2.2) | 2.0 (1.5, 2.9) | < 0.001 |
| TnI, median (IQR), ng/mL | 0.03 (0.01, 0.10) | 0.03 (0.01, 0.10) | 0.04 (0.02, 0.13) | 0.006 |
| CKMB, median (IQR), U/L | 3.7 (2.0, 10.0) | 3.6 (2.0, 9.4) | 3.7 (2.0, 10.8) | 0.809 |
| PT, median (IQR), s | 13.9 (12.3, 18.7) | 13.5 (12.0, 17.8) | 14.5 (13.0, 20.2) | 0.002 |
| APTT, median (IQR), s | 31.5 (27.2, 36.1) | 31.9 (27.6, 36.2) | 31.0 (26.0, 35.7) | 0.309 |
| **Outcomes** |  |  |  |  |
| Intubation at 28 days, No. (%) | 279 (57.3) | 165 (51.6) | 114 (68.3) | < 0.001 |
| 28-day in-hospital mortality, No. (%) | 202 (41.5) | 116 (36.3) | 86 (51.5) | 0.002 |
| Intubation or death at 28 days, No. (%) | 330 (67.8) | 199 (62.2) | 131 (78.4) | < 0.001 |
| Hospital LOS, median (IQR), mmHg | 16 (9, 26) | 16 (10, 25) | 15 (9, 27) | 0.721 |

**Supplementary Table 14: Percentage of missing data of class-defining variables in cohorts.**

|  | **Training cohort**  **(n=706)**  **Day 1** | **Validation cohort A (n=409)**  **Day 1** | **Validation cohort B (n=609)**  **Day 1** |
| --- | --- | --- | --- |
| Age | 0 | 0 | 5(0.8%) |
| Height | 5(0.7%) | 0 | 194(31.9%) |
| Weight | 0 | 0 | 241(39.6%) |
| Baseline MAP | 46(6.5%) | 6(1.5%) | 208(34.2%) |
| Baseline Total bilirubin | 166(23.5%) | 13(3.2%) | 19(3.1%) |
| Baseline SOFA | 706(100%) | 0 | 401(65.8%) |
| Baseline GCS | 706(100%) | 3(0.7%) | 367(60.3%) |
| HR | 5(0.7%) | 1(0.2%) | 281(46.1%) |
| WBC count | 35(5.0%) | 20(4.9%) | - |
| Neutrophil count | 38(5.4%) | - | 104(17.1%) |
| Lymphocyte count | 39(5.5%) | 20(4.9%) | 105(17.2%) |
| D-dimer | 167(23.7%) | 43(10.5%) | 155(37.8%) |
| SpO_2_/FiO_2_ ratio | 0 | 0 | - |
| PaO_2_/FiO_2_ ratio | 0 | 57(13.9%) | 0 |
| FiO_2_ | 0 | 1(0.2%) | 0 |
| PaCO_2_ | 4(0.6%) | 58(14.2%) | 142(23.3%) |
| Bicarbonate | 6(0.8%) | 63(15.4%) | 214(35.1%) |
| Lactate | 14(2.0%) | 109(26.7%) | 173(28.4%) |

Abbreviations: MAP, mean arterial pressure; SOFA, sequential organ failure assessment; GCS, Glasgow coma score; WBC, white blood cell.

**Supplementary Table 15: Class-defining variables before and after multiple imputation on Day 1.**

| **Variables** | **Training cohort (n=706)** | | | **Validation cohort A (n=409)** | | | **Validation cohort B (n=609)** | | |
| --- | --- | --- | --- | --- | --- | --- | --- | --- | --- |
|  | **Before imputation** | **After imputation** | ***p* value** | **Before imputation** | **After imputation** | ***p* value** | **Before imputation** | **After imputation** | ***p* value** |
| Age, median (IQR), years | 68 (54, 78) | 68 (54, 78) | 1 | 71 (64, 76) | 71 (64, 76) | 1 | 76 (66, 83) | 76 (66, 83) | 0.989 |
| Height, median (IQR), cm | 168.0 (160.0, 172.0) | 168.0 (160.0, 172.0) | 1 | 168.0 (160.0, 172.0) | 168.0 (160.0, 172.0) | 1 | 170.0 (160.0, 173.0) | 170.0 (160.0, 173.0) | 0.892 |
| Weight, median (IQR), kg | 65.0 (56.1, 75.0) | 65.0 (56.1, 75.0) | 1 | 68.0 (60.0, 75.0) | 68.0 (60.0, 75.0) | 1 | 65.0 (57.0, 75.0) | 65.0 (57.0, 75.0) | 0.346 |
| Baseline MAP, mean (SD), mmHg | 75.0 (68.3, 84.7) | 75.2 (68.7, 85.9) | 0.802 | 89.0 (82.0, 98.5) | 90.0 (82.0, 98.0) | 0.966 | 89.7 ± 15.7 | 89.6 ± 15.5 | 0.927 |
| Baseline Total bilirubin, median (IQR), µmol/L | 15.3 (9.4, 27.1) | 16.0 (9.5, 27.9) | 0.660 | 11.6 (8.4, 17.7) | 12.0 (8.8, 17.0) | 0.648 | 11.7 (8.2, 16.3) | 11.5 (8.2, 16.3) | 0.933 |
| HR, median (IQR), bpm | 100 (97, 102) | 100 (97, 102) | 0.916 | 84 (75, 95) | 84 (75, 95) | 0.861 | 94 (80, 109) | 94 (80, 108) | 0.868 |
| WBC count, median (IQR), ×10^9^/L | 10.9 (7.9, 14.5) | 10.9 (7.9, 14.4) | 0.964 | 7.7 (5.3, 10.9) | 8.1 (5.4, 10.7) | 0.847 | - | - | - |
| Neutrophil count, median (IQR), ×10^9^/L | 9.3 (6.4, 12.7) | 9.3 (6.4, 12.8) | 0.797 | - | - | - | 8.0 (5.1, 11.7) | 8.2 (5.2, 11.8) | 0.795 |
| Lymphocyte count, median (IQR), ×10^9^/L | 0.70 (0.44, 1.04) | 0.70 (0.44, 1.02) | 0.924 | 0.70 (0.49, 1.12) | 0.71 (0.50, 1.08) | 0.977 | 0.55 (0.35, 0.81) | 0.54 (0.35, 0.81) | 0.861 |
| D-dimer, median (IQR), µg/mL | 1.9 (0.8, 3.1) | 1.8 (0.8, 3.1) | 0.697 | 1.6 (0.8, 4.8) | 1.5 (0.7, 4.5) | 0.679 | 2.2 (1.1, 5.9) | 2.2 (1.0, 6.0) | 0.839 |
| SpO_2_/FiO_2_ ratio, median (IQR) | 237.3 (192.5, 256.9) | 235.2 (193.7, 253.0) | 0.461 | 234.2 (190.0, 290.9) | 234.2 (190.0, 290.9) | 0.995 | - | - | - |
| PaO_2_/FiO_2_ ratio, median (IQR), mmHg | 183.1 (135.0, 222.2) | 183.1 (135.0, 222.2) | 1 | - | - | - | 179.3 (124.3, 267.7) | 179.3 (124.3, 267.7) | 1 |
| PaCO_2_, median (IQR), mmHg | 41.5 (35.7, 47.7) | 41.6 (35.8, 47.8) | 0.925 | 35.0 (31.1, 40.9) | 35.0 (31.4, 40.9) | 0.860 | 35.0 (30.0, 42.1) | 35.0 (30.0, 42.0) | 0.914 |
| Bicarbonate, median (IQR), mmol/L | 21.2 (18.4, 24.3) | 21.6 (18.6, 24.3) | 0.305 | 23.9 (21.4, 26.5) | 23.6 (21.3, 26.4) | 0.720 | 22.2 (19.1, 25.5) | 22.4 (19.5, 26.3) | 0.344 |
| Lactate, median (IQR), mmol/L | 2.0 (1.5, 2.7) | 2.0 (1.5, 2.7) | 0.963 | 1.8 (1.3, 2.7) | 1.8 (1.3, 2.6) | 0.948 | 1.7 (1.2, 2.4) | 1.7 (1.2, 2.4) | 0.896 |

Abbreviations: MAP, mean arterial pressure; HR, heart rate; WBC, white blood cell.

**Supplementary Table 16: Clinical variables for phenotyping in different datasets.**

| **Class-defining variables** | **Training cohort (n=706)** | **Abnormal value extractor** | **Transformation** | **Validation cohort A (n=409)** | **Abnormal value extractor** | **Transformation** | **Validation cohort B(n=609)** | **Abnormal value extractor** | **Transformation** |
| --- | --- | --- | --- | --- | --- | --- | --- | --- | --- |
| Age | √ | - |  | √ | - |  | √ | - |  |
| BMI | √ | min+max | log | √ | - |  | √ | - | log |
| Baseline MAP | √ | min+max | log | √ | min+max |  | √ | min+max | log |
| Baseline Total bilirubin | √ | min+max | log | √ | min+max | log | √ | - |  |
| HR | √ | min+max | log | √ | min+max |  | √ | min+max |  |
| WBC count | √ | min+max | log | √ | min | log |  |  |  |
| Neutrophil count | √ | min+max | log |  |  |  | √ | min | log |
| Lymphocyte count | √ | min+max | log | √ | min | log | √ | min | log |
| D-dimer | √ | min+max | log | √ | min+max | log | √ | min+max | log |
| SpO_2_/FiO_2_ ratio | √ |  |  | √ | - |  |  |  |  |
| PaO_2_/FiO_2_ ratio | √ |  |  |  |  |  | √ | min+max |  |
| PaCO_2_ | √ |  | log | √ | min+max | log | √ | min+max | log |
| Bicarbonate | √ | min+max |  | √ | min+max | log | √ | min+max |  |
| Lactate | √ | min+max | log | √ | min+max | log | √ | min+max | log |

# Supplementary figures

**
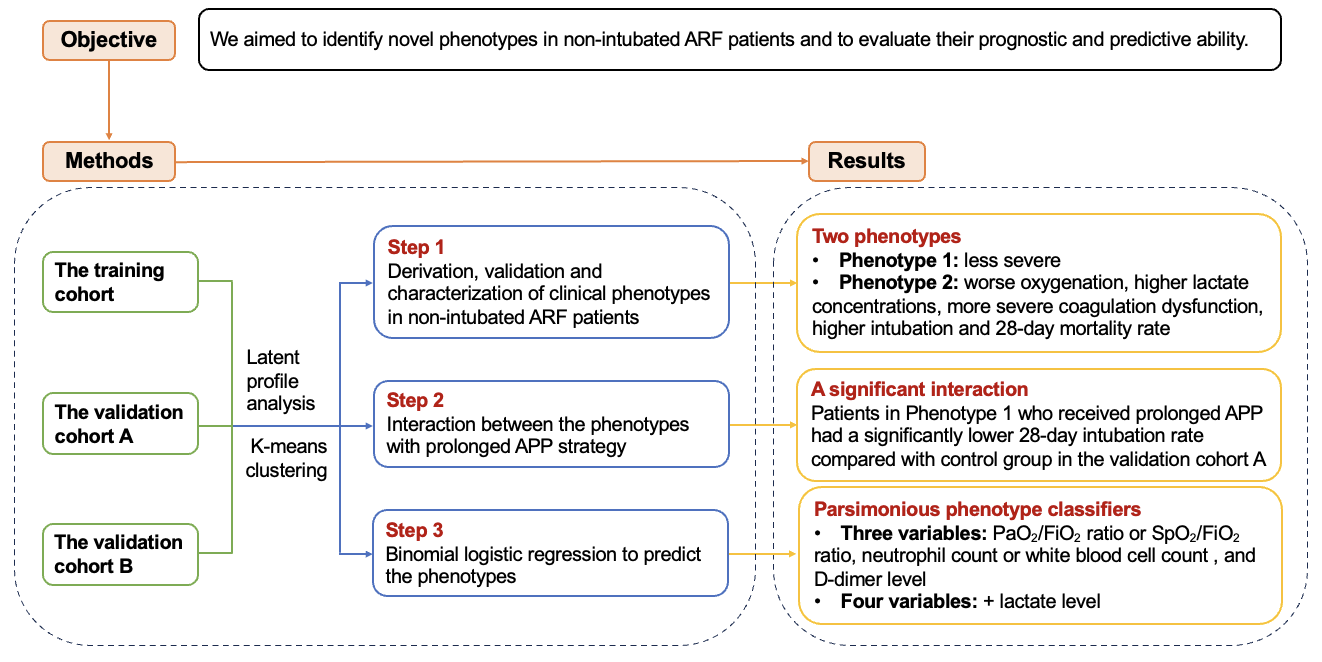
**

**Supplementary Figure 1: Schematic of study.** ARF, acute respiratory failure; APP, awake prone positioning**.**

**

**

**Supplementary Figure 2: Unsupervised k-means clustering in the training cohort showing the optimal number of clusters is k=2.**


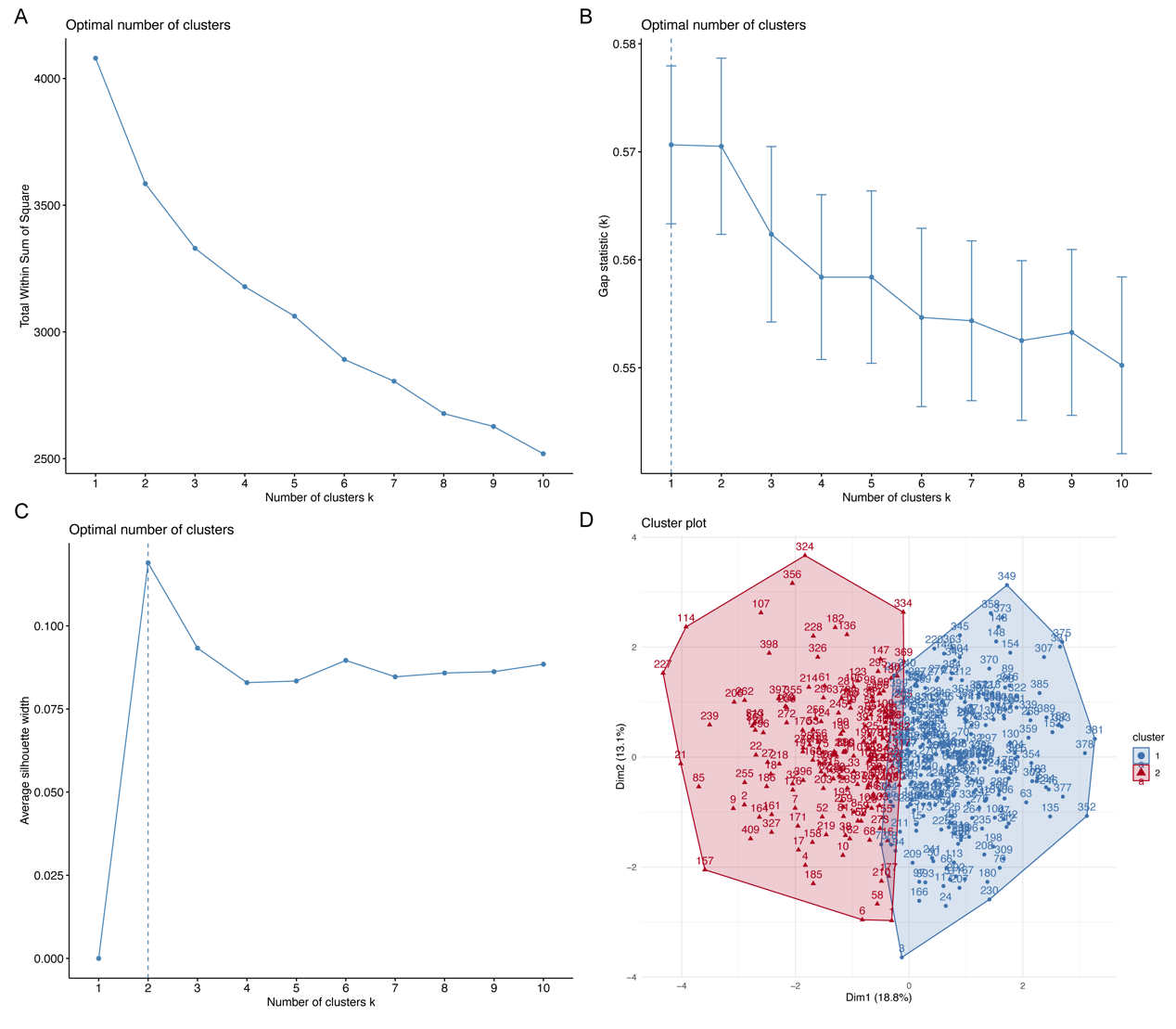


**Supplementary Figure 3: Unsupervised k-means clustering in the validation cohort A showing the optimal number of clusters is k=2.**


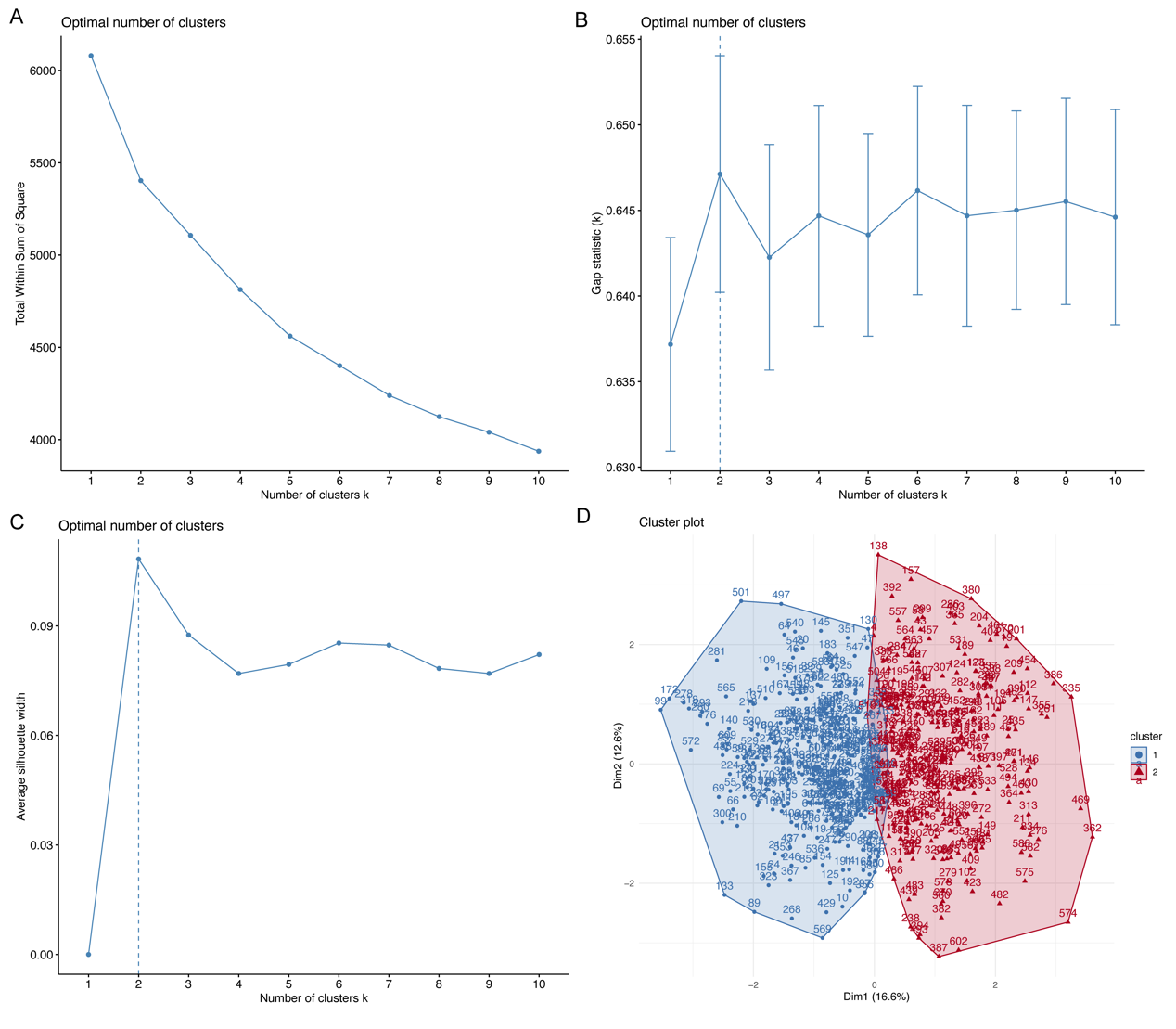


**Supplementary Figure 4: Unsupervised k-means clustering in the validation cohort B showing the optimal number of clusters is k=2.**

**Supplementary Figure 5: Comparison of variables that contribute to clinical phenotypes in the validation cohort B after excluding patients with PaO_2_/FiO_2_ above 300 mmHg.**

**
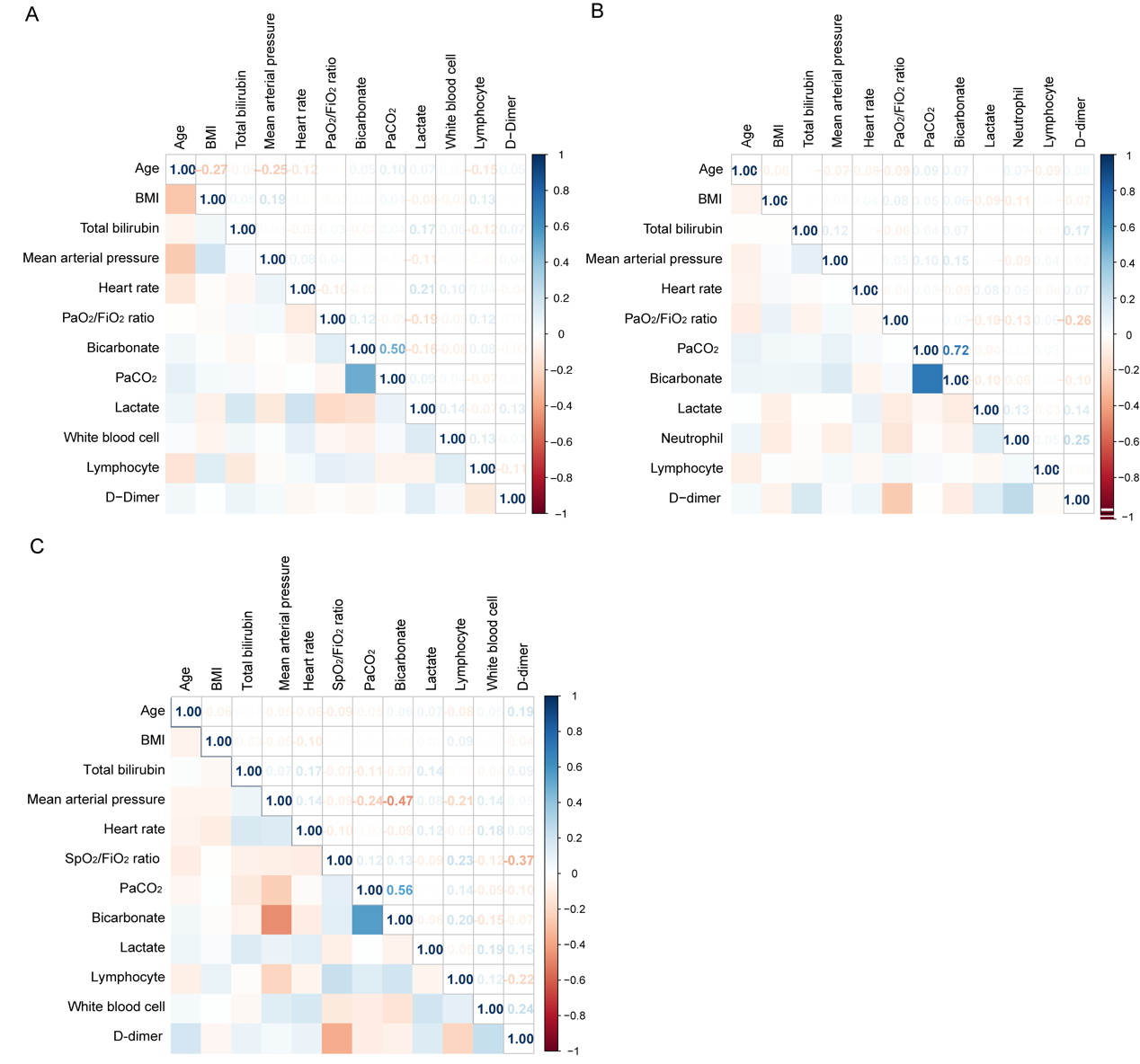
**

**Supplementary Figure 6: Heatmap of correlation between clinical variables for phenotyping.**

A: Day 1 in the training cohort, B: Day 1 in the validation cohort A, C: Day 1 in the validation cohort B.
